# Mesoporous silica-coated silver nanoparticles as ciprofloxacin/siRNA carriers for accelerated infected wound healing

**DOI:** 10.1186/s12951-022-01600-9

**Published:** 2022-08-23

**Authors:** Qiqi Liu, Ying Zhang, Jingkai Huang, Zhourui Xu, Xiang Li, Jingyu Yang, Haoqiang Huang, Shiqi Tang, Yujuan Chai, Jinbo Lin, Chengbin Yang, Jia Liu, Suxia Lin

**Affiliations:** 1 Guangdong Key Laboratory for Biomedical Measurements and Ultrasound Imaging, School of Biomedical Engineering, Health Science Center, Shenzhen University, Shenzhen, 518060 China; 2 Central Laboratory, The Second Affiliated Hospital, School of Medicine, Longgang District People’s Hospital of Shenzhen, The Chinese University of Hong Kong, Shenzhen, 518172 China; 3 Dermatology Department, Southern University of Science and Technology Hospital (SUSTech Hospital), Shenzhen, 518055 China; 4 Center of Assisted Reproduction and Embryology, The University of Hong Kong-Shenzhen Hospital, Shenzhen, 518048 China

**Keywords:** Mesoporous silica, siRNA, Synergistic therapy, Antibacterial, Skin infection, Wound healing

## Abstract

The colonization of bacterial pathogens is a major concern in wound infection and becoming a public health issue. Herein, a core–shell structured Ag@MSN (silver core embedded with mesoporous silica, AM)-based nanoplatform was elaborately fabricated to co-load ciprofloxacin (CFL) and tumor necrosis factor-α (TNF-α) small interfering RNA (siTNF-α) (AMPC@siTNF-α) for treating the bacterial-infected wound. The growth of bacterial pathogens was mostly inhibited by released silver ions (Ag^+^) and CFL from AMPC@siTNF-α. Meanwhile, the loaded siTNF-α was internalized by macrophage cells, which silenced the expression of TNF-α (a pro-inflammatory cytokine) in macrophage cells and accelerated the wound healing process by reducing inflammation response. In the in vivo wound model, the *Escherichia coli (E. coli)*-infected wound in mice almost completely disappeared after treatment with AMPC@siTNF-α, and no suppuration symptom was observed during the course of the treatment. Importantly, this nanoplatform had negligible side effects both in vitro and in vivo. Taken together, this study strongly demonstrates the promising potential of AMPC@siTNF-α as a synergistic therapeutic agent for clinical wound infections.

**Graphical Abstract:**

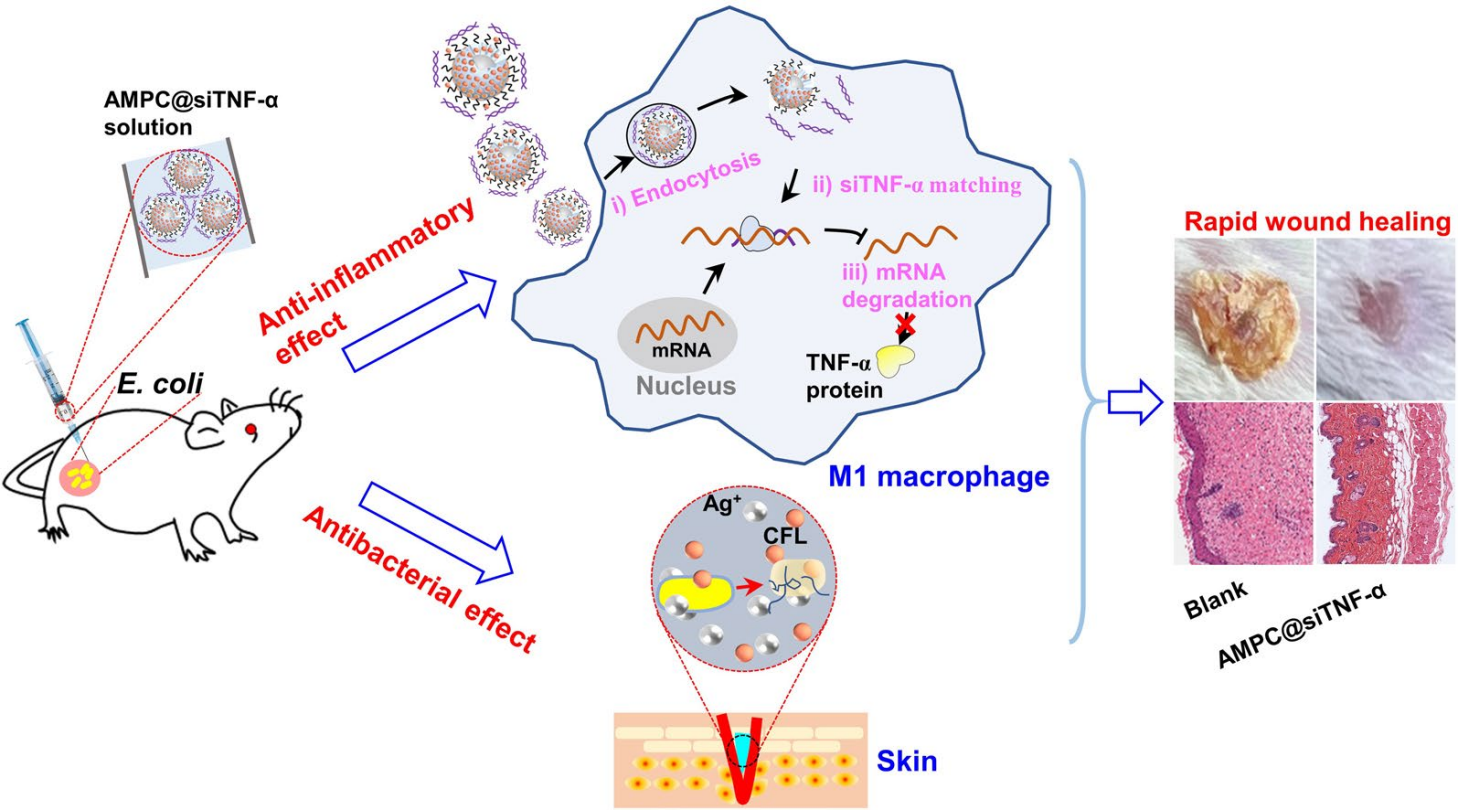

**Supplementary Information:**

The online version contains supplementary material available at 10.1186/s12951-022-01600-9.

## Introduction

Wounds could be caused by cut, burn, disease (e.g., diabetes), and surgical treatments [1, 2]. Unfortunately, these wounds can be easily contaminated by different pathogens, especially bacteria. These bacteria produce endotoxins and promote the expression of pro-inflammatory cytokines, such as tumor necrosis factor-α (TNF-α), which eventually lead to extended wound inflammation [3]. Currently, antibiotics are the major strategy for the treatment of infected wound infection [4, 5]. However, the overuse of antibiotics can cause the rapid proliferation of drug-resistance bacteria, which seriously impede the wound healing process [6]. Therefore, it is urgent to develop a novel antibacterial strategy to prevent the formation of drug-resistance bacteria and promote rapid wound healing.

With the emergence of nanotechnology, nanoparticles (NPs) are considered as promising alternatives to traditional antibiotics, owing to their bactericidal activity, excellent biocompatibility, and broad-spectrum antibacterial properties [7–10]. Among them, silver (Ag) NPs have attracted great attention due to their strong and extensive antibacterial activity [11]. However, fast ions release and poor stability limit their applications [11]. Attaching or embedding Ag NPs into organic/inorganic matrix is an excellent strategy to endow them with enhanced colloidal stability [12]. Of widely studied biocompatible nanomaterials, mesoporous silica NPs (MSNs) have been actively used as coating layers and drug reservoirs depending on their porous structure, adjustable pore size, large specific surface area, and versatile surface modification [13, 14]. Therefore, the integration of Ag NPs and MSNs (AM) can avoid the aggregation of Ag NPs and undesirable burst release of Ag, so as to effectively and safely treat wound bacterial infection. In addition, the drug loading ability of MSNs shell can be further used in synergistic antibacterial therapy. Wang et al. [15] constructed a nanoplatform of AM loaded with levofloxacin (LEVO) to treat drug-resistant bacterial infections and found that the nanoplatform could significantly reduce the infection through the synergistic antibacterial effect of Ag and LEVO. Similarly, Lu et al. [16] prepared the AM loaded with chlorhexidine (AMC), and indicated that the bacterial growth inhibition of the group treated with AMC was about 20% higher than that of the group with AM. Ciprofloxacin (CFL) is a new kind of quinolone broad-spectrum antibacterial drugs for Gram-positive and negative bacteria [17]. And CFL has a zwitterionic molecular structure and can be loaded into the MSNs through electrostatic interaction [18]. Accordingly, it was believed that AM loaded with CFL would have the potential synergistic antibacterial properties.

In the stage of wound inflammation, the expression of some pro-inflammatory cytokines, especially TNF-α, is up-regulated [19]. Some studies have confirmed that high levels of TNF-α were closely related to the wound microenvironment, which could directly lead to the emergence of chronic wounds [20–22]. Considering the adverse effects of antibiotics, gene therapy, as a safe and effective method, has gradually attracted people’s attention. Small interfering RNA (siRNA) is an important gene-silencing technique in gene therapy, which can specifically knock down the expression of target genes by mediating the degradation of target mRNA [23–25]. *TNF-α* gene can be silenced by a synthetic siRNA with complementary sequences. For example, intestinal inflammation was alleviated by delivering siTNF-α to inhibit TNF-α expression [26]. However, some factors limit the biomedical utility of the synthetic siRNAs, such as the negative charge, instability in the blood circulation, and immunogenicity [27, 28]. Therefore, it is urgent to exploit an ideal vector that can protect siRNA from degradation and inhibit the TNF-α gene’s expression to achieve a better wound healing effect.

**Scheme 1:**
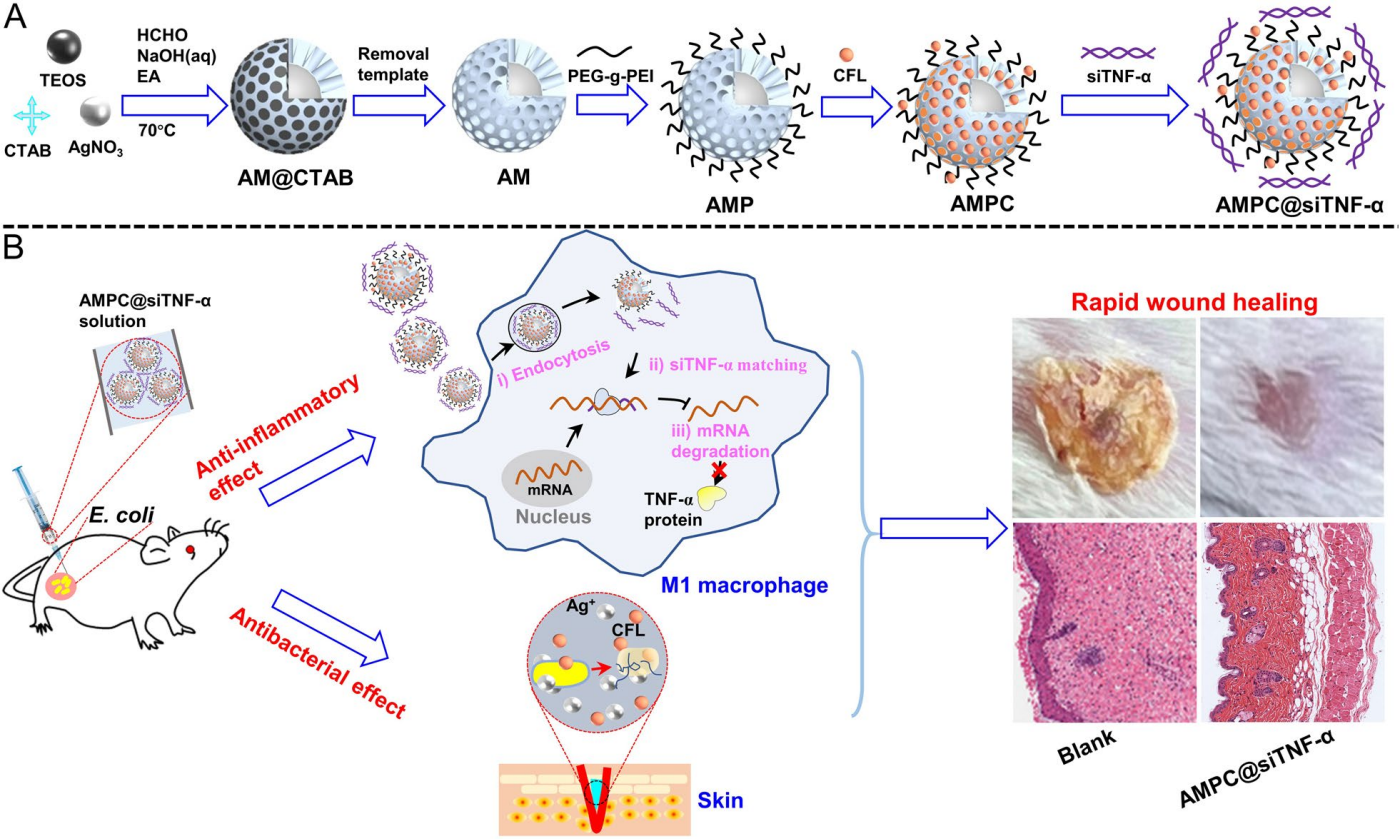
Schematic illustration of the multifunctional nanoplatform of AMPC@siTNF-α and its application for synergistically promoting wound healing. **A** The preparation of AMPC@siTNF-α. AM was prepared by the one-pot method and modified with PEG-g-PEI biocompatible polymer. And then, CFL and siTNF-α were loaded into these nanoparticles (NPs). **B** The application of AMPC@siTNF-α for synergistic therapy of *E. coli* infected wound in vivo. After the NPs were applied to the site of wound infection, the released siTNF-α down-regulated the expression of TNF-α through the RNA interference mechanism. In addition, the released Ag^+^ and CFL could synergistically inhibit the growth of *E. coli,* thus accelerating would healing process

In this work, Ag@MSN encapsulated with PEG-g-PEI (AMP) was prepared to load CFL and siTNF*-α* (AMPC@siTNF-α) for promoting wound healing by synergistic inhibition of bacterial proliferation (Scheme 1). In vitro, the released CFL and silver ions (Ag^+^) from AMPC@siTNF-α could enhance the bactericidal effect. The intracellular siTNF-α could down-regulate *TNF-α* expression of macrophage cells, which was expected to inhibit the pro-inflammatory response. In vivo*,* the fabricated antibacterial nanoplatform showed excellent bacteria-killing activity, promoting wound healing, and low biotoxicity in an *E. coli*-infected mouse wound model. Therefore, the multifunctional nanoplatform, AMPC@siTNF-α, might be a promising wound dressing for skin infection treatment depending on the synergistically bacterial-killing effect.

## Materials and methods

### Materials

Cetyltrimethylammonium bromide (CTAB), absolute ethanol, silver nitrate solution (AgNO_3_, ≥ 99.0%), formaldehyde (HCHO), branched PEG-g-PEI, ribonuclease A (RNase A), and 4′,6-diamidino-2-phenylindole (DAPI) were purchased from Sigma-Aldrich Co., Ltd (St. Louis, MO, USA). Sodium hydroxide solution (NaOH), formaldehyde solution (37%), ethyl acetate (EA), and tetraethyl orthosilicate (TEOS) were obtained from Macklin Co., Ltd (Shanghai, China). Ammonium nitrate (NH_4_NO_3_) and CFL were from Aladdin Reagent Co., Ltd (Shanghai, China). Dulbecco’s modified eagle’s medium (DMEM), luria–bertani (LB) broth, penicillin–streptomycin solution, phosphate-buffered saline (PBS), fetal bovine serum (FBS), and lipofectamine™ 3000 (Lipo3000) were purchased from Gibco (Thermo Fisher, USA). Cell counting kit-8 (CCK-8) and lipopolysaccharide (LPS) were from Biosharp Co., Ltd (Beijing, China). siTNF-α, Cy3-siRNA-negative control (Cy3-siNC) was synthesized by Shanghai GenePharma Co., Ltd. (Shanghai, China). Trypsinization (0.25%, without EDTA) was obtained from Solarbio Biotech Co., Ltd (Beijing, China). TRIcom reagent was from TIANMO BIOTECH Co., Ltd (Beijing, China). Evo M-MLV RT kit was purchased from Accurate Biology Co., Ltd (Hunan, China). Stormstar Sybr Green qPCR Master Mix was from DBI Bioscience Co., Ltd (Shanghai, China). Mouse TNF-α ELISA kit was purchased from Abcam (ab208348, UK). *Escherichia coli* (*E. coli*, CMCC44103) and *Staphylococcus aureus* (*S. aureus*, ATCC6538P) were obtained from the China General Microbiological Culture Collection Center. RAW 264.7 cell was purchased from American Type Culture Collection (ATCC, Manassas, USA) and it was an immortalized mouse myoblast cell line and could be activated to initiate M1 polarization, releasing inflammatory factors, including TNF-α [29].

### Preparation of PEG-g-PEI-modified mesoporous silica-coated silver (AMP)

AM was prepared as described Song et al. [30] and Wang et al. [15] with minor modifications. Firstly, 0.3 mL of NaOH aqueous solution (2 M) and 0.1 g of CTAB were added to 50 mL of deionized water for incubation at 37 °C for 30 min. And then, 0.3 mL of HCHO solution (1 M) and 1 mL of AgNO_3_ solution (0.1 M) were added under stirring. Subsequently, 0.5 mL of TEOS was dropped into the reactive mixture. All the ingredients were then continuously stirred at 80 °C and refluxed for 2 h. The resultant precipitate was collected via centrifugation at 8000 rpm for 10 min and washed with ethanol for three times. In order to remove the surfactant template CTAB, 0.06 g of NH_4_NO_3_ was added to the NPs dispersed in 60 mL of ethanol solution under a sonic bath for 2 h. After drying for 120 min at 60 °C, the AM was obtained without the template. And then, 10 mg of AM was dissolved in 10 mL of deionized water, and 0.5 mL of PEG-g-PEI solution (100 mg/mL) was dropped into the solution under stirring (300 rpm/min, 25 °C) overnight. Finally, AMP (1 mg/mL) was obtained through centrifuging at 12,000 rpm/min for 15 min, discarding the supernatant, and washing the precipitation with deionized water for three times to remove the excess PEG-g-PEI. Their morphological properties were detected by transmission electron microscopy (TEM, HT7700, Hitach, Ltd).

### Ciprofloxacin loading by AMP (AMPC)

In order to load CFL into drug carriers, AMP (5 mg) was mixed with CFL aqueous solution (500–4000 μg/mL, 5 mL) under stirring at 25 °C. Then, the mixture was separated by centrifugation (8000 rpm/min, 5 min) and washed several times until there was no free CFL in the supernatant. The amount of free CFL in the supernatant was calculated from a calibration curve based on the absorbance intensity at 275 nm by UV–vis (TP-720 spectrometer, Tianjin Tuopu Instrument Co., Ltd). The percentage of CFL loading into AMP was calculated as follows:

$$\text{LE }\left(\%\right)\text{ } = \frac{{\text{m}}_{\text{oriCFL }}-{\text{m}}_{\text{supCFL}}}{{\text{m}}_{\text{AMP}} \, + \, {\text{m}}_{\text{oriCFL}}} \, \times \, {100}\%$$

where the m_oriCFL_, m_supCFL,_ and m_AMP_ represent the mass of original CFL, CFL in the supernatant, and AMP, respectively. The LE represents the loading efficiency.

### Drug release from AMPC

To detect the release of CFL from AMPC, the AMPC (2 mg) were dispersed in PBS (pH 7.4, 2 mL) and transferred into a dialysis bag with a molecular weight cut-off of 1000 Da and kept in PBS (50 mL) on a shaking table at 37 °C for 48 h. After 2 mL of the solution was removed at different time points, the drug release efficiency was measured by UV–vis at 275 nm. In order to keep the solution volume constant, 2 mL of fresh PBS needed to be added after each sampling.

To study the release of Ag from AMPC, the AMPC was suspended in an LB culture medium. After the mixture was incubated at 37 °C, the UV–vis adsorption of the AMPC solution was monitored over a time period. The amount of consumed Ag was detected at 417 nm using a microphone reader (Bio-teak, Epoch-2).

### Preparation and characterization of AMP loaded with siRNA (AMP@siRNA)

First, AMPC was obtained according to the method described above and then AMP or AMPC and siNC (sense: 5′-CGAAGUGUGUGUGUGUGGC-3′, antisense: 5′-GCCACACACACACACUUCG-3′) with different weight ratios (0:1, 7.5:1, 15:1, 30:1, 60:1, and 120:1) were mixed at 25 °C for 30 min. And then the binding capacity was evaluated by the agarose gel electrophoresis (110 V, 8 min), the gel was imaged under a UV transillumination (FlourChem E, ProteinSimple, San Jose, CA, USA) and the gray value was calculated by Image J (Bethesda, Maryland, USA). The zeta potential and hydrodynamic diameter of AMP@siNC were then measured by Zetasizer Nano-ZS90 (Malvern Panalytical, Ltd).

### Serum enzymatic protection test

To determine the ability of AMP to protect siRNA from RNase A, the AMP and siNC (weight ratio of 15:1) were incubated at a 2 μL of RNase A (0.5 μg/mL) for 0, 6, 12, 18, 24, and 30 min respectively. Subsequently, the solution was mixed with 1% SDS at 4 °C for 3 min. Then the remaining siRNAs were detected by agarose gel electrophoresis (110 V, 8 min) and quantified based on the fluorescence intensity.

### The cytotoxicity and hemolysis assay of AMP

To evaluate the cytotoxicity of AMP in vitro, 100 μL of RAW 264.7 cells with a density of 5000 cells/well were seeded into 96-well plates. After culturing for 24 h, AMP with different concentrations (5, 10, 20, 40, 60, 80, 100, 120, and 140 ppm) were placed in the wells and co-cultured for another 24 h. Then, the culture medium was removed, and the wells were washed twice with PBS. For each well, 10 μL of CCK-8 solution and 90 μL of culture medium were added, and the plate was incubated in an incubator (37 °C, 5% CO_2_) for 1 h. Subsequently, the cell viability was measured at the absorbance of 450 nm by a microplate reader (Bio-teak, Epoch-2) and calculated according to the following formula:

$$\text{Cell viability }\left(\%\right)\text{ = }\frac{{\text{A}}_{\text{eg}}-{\text{A}}_{\text{bg}}}{{\text{A}}_{\text{ng}}-{\text{A}}_{\text{bg}}} \, \times \, {100}\%$$

where A_bg_ and A_ng_ represent the absorbance of cell- and AMP-free medium with CCK-8 solution, respectively. A_eg_ represents the absorbance of medium with cells, CCK-8, and AMP solution.

To investigate the hemolytic effects of AMP to red blood cells (RBCs), 500 μL of blood was diluted tenfold with PBS. The blood was mixed gently and centrifuged at 10,000*g* for 5 min. The supernatant was discarded, and RBCs were washed a few times by suspending them in a PBS solution (pH 7.4) until the supernatant was clear. Finally, RBCs were resuspended with 10 mL of PBS. To evaluate the hemolytic effects, 200 µL of RBCs were incubated with 800 µL of H_2_O (as positive control), 800 µL of PBS (as negative control), and AMP with different concentrations for 4 h in a 37 °C incubator. After incubation, the samples were further centrifugated at 10,000*g* for 5 min, and 100 µL of supernatants were extracted to quantify hemoglobin by recording the absorbance at 577 nm. The percentage of hemolysis rate was calculated as follows.

$$\text{Hemolysis rate }\left(\%\right)\text{ = }\frac{{\text{A}}_{\text{sam}}-{\text{A}}_{\text{neg}}}{{\text{A}}_{\text{pos}}-{\text{A}}_{\text{neg}}} \, \times \text{ 100}\%$$

where the A_sam_, A_neg_, and A_pos_ represent the absorbance value of treatment, negative and positive groups, respectively.

### siRNA transfection

RAW264.7 cells were cultured in DMEM medium supplemented with 10% FBS, 1% penicillin (100 μg/mL), and streptomycin (100 μg/mL) in an atmosphere with 5% CO_2_ at 37 °C. Subsequently, RAW264.7 cells were seeded onto 24-well plates with a density of 3 × 10^4^ cells/well, and cultured for 24 h. And then, cells were activated with 1 μg/mL of LPS. After 4 h, the maintenance medium was replaced with serum-free DMEM. Meanwhile, the AMP (1 mg/mL) and Cy3-siNC (100 pM) were mixed at a weight ratio of 15:1 and 30:1 at 25 °C for 40 min. Then, the above AMP@siNC were added to the 24-well plates and incubated for 4 h.

To examine the uptake efficiency, these cells were imaged using fluorescent microscopy and assessed by flow cytometry, respectively. Additionally, to study the gene *TNF-α* expression, some cells were cultured for 72 h post-transfection in DMEM medium with 10% FBS after removing the old medium-containing material. The sense and antisense sequences of siTNF-α were listed as follows: sense: 5′-GUCUCAGCCUCUUCUCAUUdTdT-3′, antisense: 5′- AAUGAGAAGAGGCUGAGACdTdT-3′.

### Fluorescence imaging and siRNA transfection efficiency

After being treated with AMP@siNC for 4 h, cells were washed three times with PBS (pH 7.4) and fixed with 4% formaldehyde for 15 min. Cells were then stained with DAPI for 20 min. The filters of the inverted microscope were set for DAPI (excitation at 405 nm and the emission was collected with a 450/50 nm band pass filter) and Cy3 (excited with 543 nm and emission was collected with a band pass filter 605/50 nm). To quantify cell internalization, the post-transfection cells were washed three times with PBS and collected by trypsinization (0.25%, without EDTA). Cy3 was used as a fluorescent marker (filter set for ECD was applied) to quantify the fluorescence intensity. The samples were evaluated by a flow cytometer (CytoFLEX, Beckman).

### In vitro anti-inflammatory activity

To demonstrate the anti-inflammatory activity, LPS-activated macrophages were used to elicit the release of the inflammatory mediator TNF-α [31, 32]. The transcription level of *TNF-α* gene was investigated by qRT-PCR according to previous experiences [33]. In brief, the total RNA from RAW264.7 cells treated with AMP, AMP/siNC, AMPC, AMP/siTNF-α, and Lipo3000/siTNF-α was extracted using a TRIzol reagent (Invitrogen) and quantified using a micro-spectrophotometer (Epoch2, Biotek Instruments). Total RNA (800 ng) was reverse-transcribed to cDNA using PrimeScriptTM RT reagent Kit (AG11705, Aikerui Biological Engineering Co., Ltd, Hunan, China). The mRNA level of *TNF-α* gene was measured by qRT-PCR using the SYBR green dye (DBI-Bioscience 2143) in a QuanStudio 1 applied biosystem. The qRT-PCR was performed in a 20 µL reaction volume containing SYBR Premix Ex Taq II (10 µL), forward prime (10 µM, 0.8 µL), reverse primer (10 µM, 0.8 µL), cDNA template (5 ng/µL, 2 µL), and ddH_2_O (6.4 µL). The PCR conditions were denaturation at 95 °C for 30 s, followed by 40 cycles of amplification (95 °C for 5 s, 60 °C for 30 s). The melting curves were measured at 95 °C for 5 s and 60 °C for 1 min. The *β-actin* gene was used as the internal control reference gene. Finally, gene expression was calculated using the 2^−∆∆Ct^ method [34]. The primer sequences were as follows: β-actin F: 5′-GGTCATCACCATTGGCAATG-3′, R: 5′-TAGTTTCGTGGATGCCACAG-3′; TNF-α F: 5′-GTCTGGGCAGGTCTACTTTGG-3′, 5′-GGTTGAGGGTGTCTGAAGGAG-3′.

Furtherly, TNF-α content in the cell-free supernatants was determined using the TNF-α ELISA kit according to the manufacturer’s instructions. Briefly, 50 μL of the antibody cocktail was added to each well with 50 μL of samples, then sealed and incubated for 1 h on a plate shaker (25 °C, 400 rpm/min). Subsequently, each well was washed with 350 μL of 1× washing buffer PT for 3 times, then added 100 μL of TMB development solution and incubated for 10 min on a plate shaker (400 rpm/min) in the darkness. Finally, 100 μL of stop solution each well was added and shaked for 1 min. And the OD value was measured by UV–vis at 450 nm.

### In vitro antibacterial activity

The minimum inhibitory concentrations (MICs) of the different NPs for *E. coli* and *S. aureus* were determined by a micro broth dilution method. The strains were cultured in LB medium at 37 °C to the logarithmic phase. And then, the bacterial fluid was diluted to a concentration of 5 × 10^5^ colony-forming units per mL (CFU/mL). Subsequently, AM, AMP, AMPC, and AMPC/siTNF-α were separately added into tubes with 4 mL of bacterial cultures and shaken for 24 h at 37 °C. After naked eye observation, the lowest concentration of the NP in the tube without bacteria growth was determined as MIC.

To further evaluate the antibacterial activity of these NPs, *E. coli* and *S. aureus* in the exponential phase were serially diluted with LB medium to a concentration of 5 × 10^5^ CFU/mL. Then, the bacterial suspension was added to 96-well plates and treated with AM, AMP, AMPC, and AMPC/siTNF-α (50 μg/mL). At different time intervals, the OD_600_ of bacterial suspensions was determined using a microphone reader (Bio-teak, Epoch-2) to obtain killing curves. Additionally, after incubation at 37 °C for 12 h, 10 µL of the diluted bacterial solution was spread on LB agar plates. After incubation at 37 °C for another 12 h, digital images of each plate were captured, and the CFU/mL and antibacterial ratio were obtained. CFU/mL was calculated according to the following equation:

$$\text{CFU/mL = }\frac{\text{colony number }\times \text{ dilution ratio}}{\text{plated volume}}$$

In addition, the combination effect of CFL and Ag was evaluated through combination index (CI) analysis according to the following equation [15]:

$$ {\text{CI }} = \, {{{\text{D}}_{{1}} } \mathord{\left/ {\vphantom {{{\text{D}}_{{1}} } {{\text{D}}_{{{\text{CFL}}}} }}} \right. \kern-\nulldelimiterspace} {{\text{D}}_{{{\text{CFL}}}} }} + {{{\text{D}}_{{2}} } \mathord{\left/ {\vphantom {{{\text{D}}_{{2}} } {{\text{D}}_{{{\text{Ag}}}} }}} \right. \kern-\nulldelimiterspace} {{\text{D}}_{{{\text{Ag}}}} }} + {{{\text{D}}_{{1}} {\text{D}}_{{2}} } \mathord{\left/ {\vphantom {{{\text{D}}_{{1}} {\text{D}}_{{2}} } {{\text{D}}_{{{\text{CFL}}}} {\text{D}}_{{{\text{Ag}}}} }}} \right. \kern-\nulldelimiterspace} {{\text{D}}_{{{\text{CFL}}}} {\text{D}}_{{{\text{Ag}}}} }} $$

where D_CFL_ represents the dose at which CFL produces MIC effect alone and D_1_ is the dose of CFL required to produce the same MIC effect in combination with Ag; similarly, D_Ag_ is the dose of Ag required to produce MIC effect alone and D_2_ is the dose of Ag required to produce the same MIC effect in combination with CFL. It is considered as synergism (CI < 1), antagonism (CI > 1), and additive effects (CI = 1).

### In vivo wound healing and safety evaluation

The in vivo antibacterial efficacy of AMPC@siTNF-α was examined on the *E. coli* infection model in terms of wound recovery and histological analysis. All experiments involving animals were approved by the Institutional Animal Ethical Committee at the Laboratory Animal Research Center at Shenzhen University (Shenzhen, China, approval number: AEWC-202200012). Briefly, 6–8-week-old BALB/c mice (18–22 g) were obtained from Guangdong Medical Laboratory Animal Center (Guangdong, China). Mice were anesthetized by intraperitoneal injection of 4% pentobarbital sodium (1.0 mL/kg). Round skin wounds were created on the back with a biopsy puncture of 8 mm diameter, and then 10 μL of *E. coli* suspension (10^7^ CFU/mL) was added to the wound surface. One day later, the mice were randomly divided into 8 groups (n = 4), 200 μL of AM, AMP, AMPC, AMP@siTNF-α, and AMPC@siTNF-α suspensions (50 μg/mL) and CFL solution (35 μg/mL) in PBS were placed on the wounds. The wounds were treated with 200 μL of PBS and levofloxacin (LEVO, 60 μg/mL) as the negative and positive controls, respectively. The area and images of the wound were recorded from 0 to 12 days. After 12 days of treatment, wound tissues were collected and dipped in fixative (4% paraformaldehyde). Wound tissues were sectioned and stained at Wuhan Service Biotechnology Co., Ltd., and the images were then recorded and analyzed using a Pathology Sectioning Scanner (LEICA-Aperio, carbon disulfide).

### Statistical analysis

All experiments were conducted at least three times, and the data were shown as mean ± standard deviation (SD). *T*-test were used to evaluate the significance of different data. It was considered as statistically significant when *p* < 0.05 (*), *p* < 0.01 (**), and *p* < 0.001 (***).

## Results and discussion

### Preparation and characterization of AMPC

**Fig. 1:**
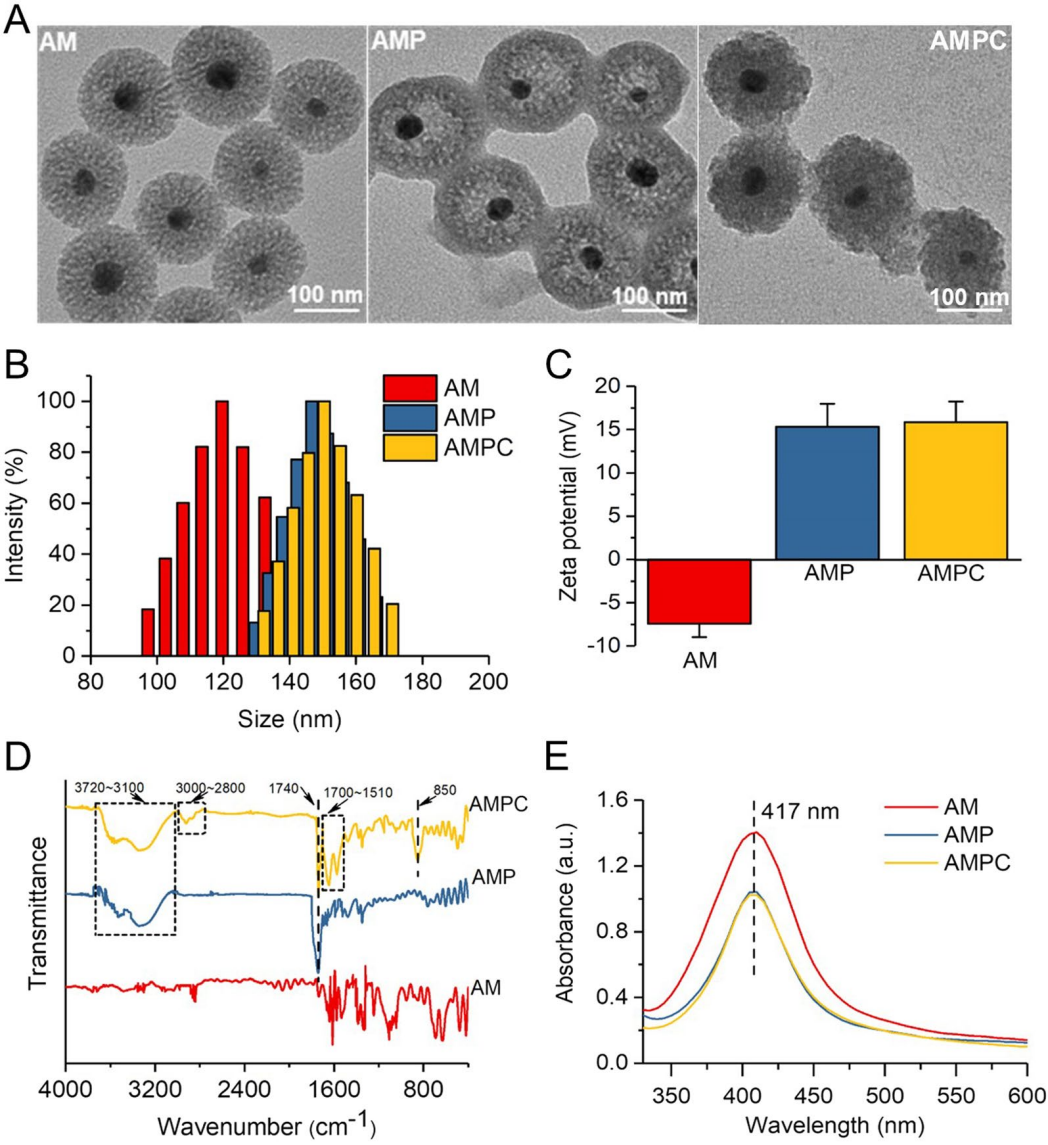
Characterization of AMPC. **A** TEM images, **B** the hydrodynamic size, **C** the zeta potential, **D** the fourier transform infrared (FTIR) spectrum, and **E** the UV–vis absorption spectrum of AM, AMP, and AMPC

According to previous reports [35–37], AMP was obtained by modifying AM with PEG-g-PEI and then CFL could be loaded into AMP. The prepared AM showed a uniform and monodispersed spherical morphology with a typical porous core–shell structure, in which Ag was embedded in the center of the mesoporous silica shell layer. Compared with the morphology of AM, some things modified on the surface of AMP and AMPC could be clearly observed in Fig. 1A. The dynamic light scattering (DLS) results indicated the hydrodynamic diameters of AM, AMP, and AMPC were 123 nm, 146 nm, and 147 nm, respectively (Fig. 1B). The narrow size distribution indicated that these NPs had excellent size uniformity. Besides, the zeta-potential of those NPs was also determined. After modification of PEG-g-PEI and CFL, the zeta-potential of AM changed from − 7.42 mV to + 15.32 mV and + 15.86 mV respectively (Fig. 1C). Furthermore, the fourier transform infrared (FTIR) spectrum was employed to confirm the chemical structure of those NPs. As shown in Fig. 1D, the FTIR of AMP possessed a characteristic peak of 1740 cm^−1^ (PEG-g-PEI, C=O stretching vibration of amide peak) and a double peak of 3720–3100 cm^−1^ (PEG-g-PEI, NH_2_ stretching vibration), suggesting AM was modified by PEG-g-PEI. For the FTIR of AMPC, in addition to the characteristic peak of AMP, it also had a broad absorption peak at 3000–2800 cm^−1^ (OH stretching vibration of carboxylic acid) and a double peak of 1700–1510 cm^−1^ (CFL, skeleton characteristic vibration peak of benzene ring) and a characteristic peak of 850 cm^−1^, indicating that CFL was successfully loaded into AMP. In addition, the absorption peaks of AM, AMP, and AMPC in the UV–vis spectrum were at about 417 nm (Fig. 1E). The changes in morphology, average size, zeta-potential, and FTIR indicated that PEG-g-PEI has been successfully decorated on AM surface and CFL was loaded into AMP.

### The loading and release of ciprofloxacin and silver ion

**Fig. 2:**
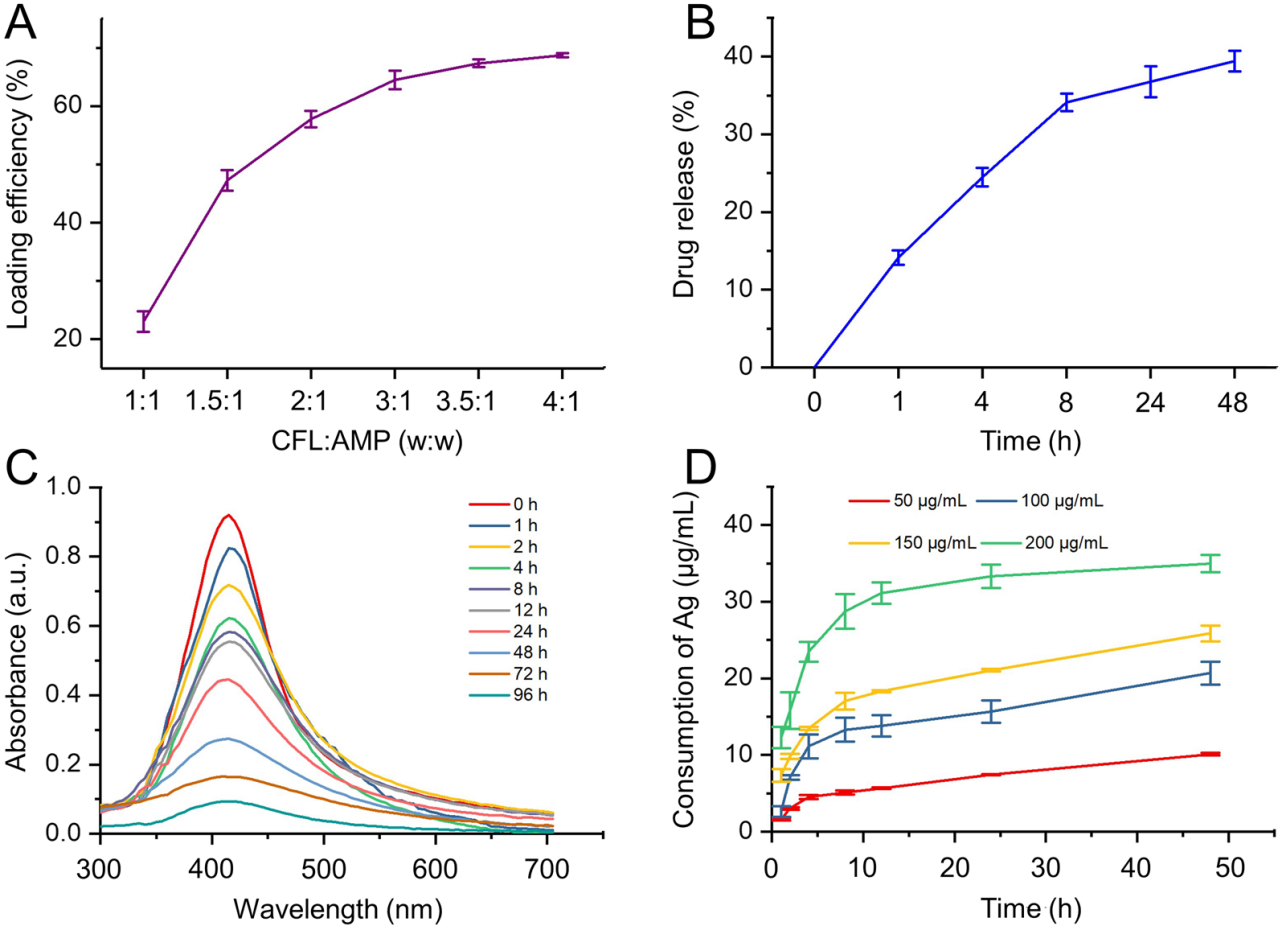
The loading and release profiles of antibacterial components. **A** The loading efficiency of ciprofloxacin (CFL) of AMP at different weight ratios. **B** Cumulative release profiles of CFL from AMPC in PBS (pH 7.4) at 37 °C. **C** The UV–vis spectra and **D** consumption of Ag from AMPC with different concentrations over time in LB medium at 37 °C

The loading efficiency and release behavior of CFL in AMP were carefully evaluated by UV–vis. The pure CFL displayed a characteristic absorption peak at 275 nm. The intensity of the absorption peak of CFL increased, accompanied by the higher concentration of CFL (Additional file 1: Fig. S1A, B). In order to obtain the encapsulation and loading rates of CFL, AMP was mixed with CFL at different mass ratios. When the mass ratio of CFL and AMP was between 1:1 and 3:1, the encapsulation and loading efficiencies of CFL increased significantly from 22 to 65%, 23–64%, respectively. When CFL: AMP (w:w) was 4:1, the maximum encapsulation and loading efficiencies both reached the maximum value of 69% (Additional file 1: Fig. S2, Fig. 2A), which was higher than that of Au NPs loaded-CFL (60%, 34%) [38] and fibrin NPs loaded-CFL (52%, 0.59%) [39].

The release behaviors of CFL from AMPC in vitro were also tested in PBS (pH 7.4) at 37 °C for 48 h. A rapid release happened in the first 8 h, reaching 34%. In the following stage, a prolonged release profile occurred in the next 8 h to 48 h and stabilized after 48 h, with a maximum release percentage of 41% (Fig. 2B). The sustained and controlled release profiles of CFL was in support of the viewpoint that porous core–shell NPs could effectively control the release of drugs [40, 41].

The release profiles of Ag from AMPC in vitro were performed indirectly by culturing AMPC in an LB medium at 37 °C. The UV–vis spectrum showed that the absorption peak intensity at 417 nm gradually decreased over time (Fig. 2C). The change of Ag^+^ content was determined by standard curve UV–vis absorbance (Additional file 1: Fig. S1C). The cumulative consumption of Ag from AMPC was improved along with the increase of the NP concentration. When the concentration of AMPC was 200 μg/mL, Ag release increased rapidly in the first 12 h and then remained saturated in the following 12 to 50 h, with a maximum release of 35 μg/mL (Fig. 2D). This may be attributed to the oxidative chelation process, which converted AMPC into Ag^+^ through various salts and peptides in the LB medium [15, 42]. Moreover, some peptides (e.g., glutathione) are also common in natural bacterial biofilms, which will enhance the release of Ag^+^ in the infectious wound environment [43, 44].

### Preparation and characterization of AMP@siRNA

**Fig. 3:**
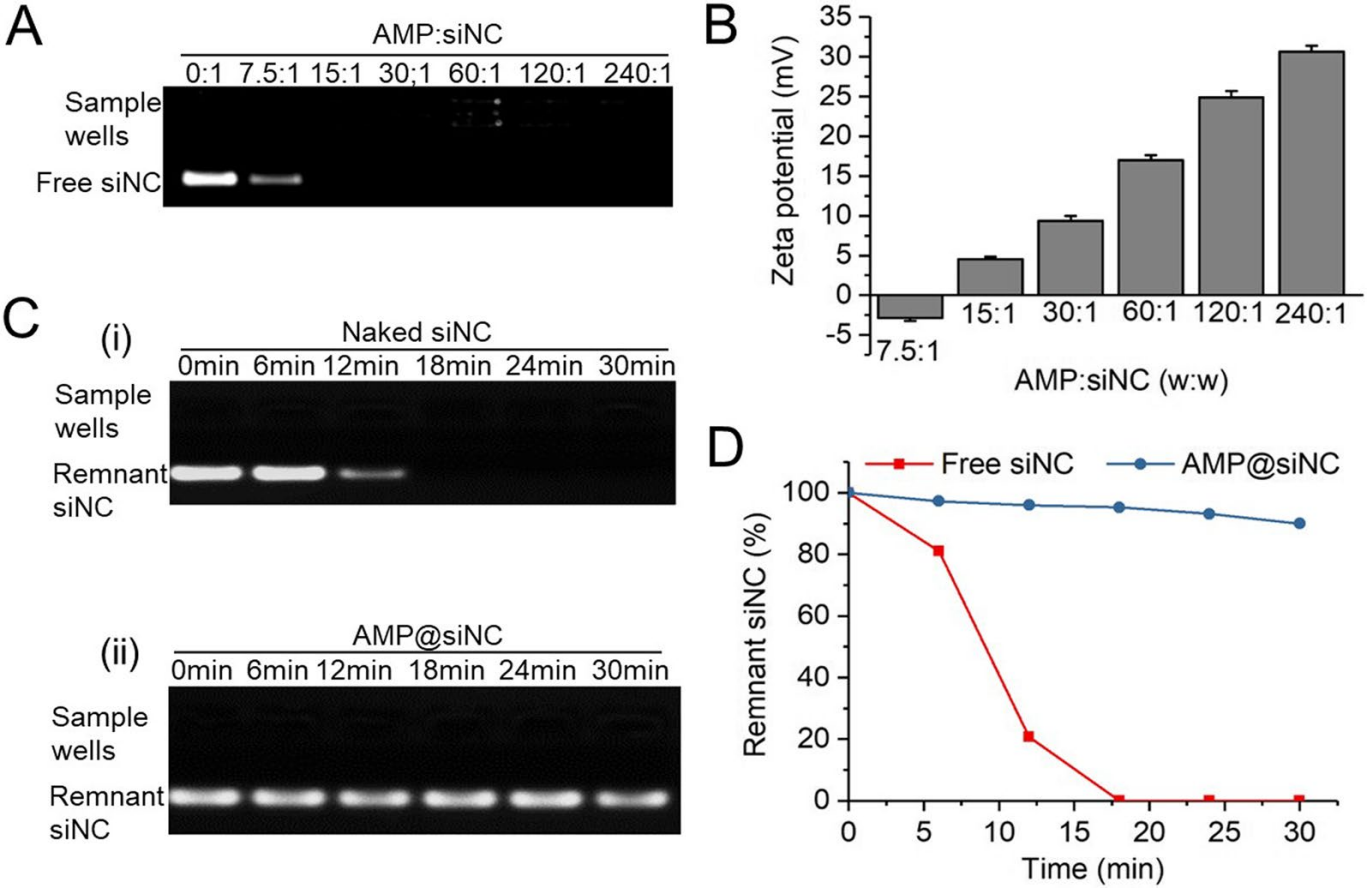
Characterization of AMP@siRNA. **A** Electrophoretic mobility and **B** zeta potential of AMP@siNC at different weight ratios. **C** Agarose gel electrophoresis results of remnant siNC. The degradation of: (i) naked siRNA, and (ii) AMP@siNC (15:1) after incubation with RNase A-containing solution for a predetermined time to obtain the remnant siNC. **D** Quantitative analysis of remnant siNC of free siNC and AMP@siNC after incubation with RNase A-containing solution for a predetermined time by Image J software

The siRNA loading efficiency was evaluated by agarose gel electrophoresis. The results indicated that when the weight ratio of AMP or AMPC and siNC went up to 15:1, no free siRNA was observed in the agarose gel, meaning that all siRNA had been retarded by AMP or AMPC in the sample wells (Fig. 3A, Additional file 1: Fig. S3). Subsequently, the surface zeta potential of AMP@siNC gradually rised with an increased weight ratio of AMP and siNC (Fig. 3B). The zeta potential of AMP@siNC was reversed from − 2.87 ± 0.35 mV (weight ratio at 7.5:1) to 30.63 ± 0.74 mV (weight ratio at 240:1), which would help the loaded siRNA cross the negatively charged cell membrane to the cytoplasm [45]. As a siRNA carrier, it is important to protect siRNA from serum nuclease degradation. Firstly, siNC was incubated in deionized water containing RNase A for a different time at 37 °C, and the results showed that the brightness of the remnant siNC bands gradually darkened with extended incubation time. When the incubation time was 18 min, the brightness disappeared completely, indicating that siNC was completely degraded by RNase A (Fig. 3Ci). Then, the AMP@siNC (weight ratio at 15:1) were incubated at the same concentration of RNase A solution for the same time, followed by the separation of loaded siNC from AMP@siNC using SDS. Results indicated that siNC had no noticeable degradation under the protection of AMP (Fig. 3Cii). In addition, quantitative analysis of siNC degradation revealed that naked siNC was obviously degraded by RNase A, and AMP could well protect siNC from degradation (Fig. 3D). These results indicated that AM could be protonated by PEG-g-PEI to form AMP, which could carry siRNA and protect siRNA from degradation.

### Biosafety evaluation of AMP

It is necessary to evaluate NPs biosafety for their biological application. In the study, the toxicity of AMP was tested by co-culturing with RAW264.7 cells. Results indicated approximately 90% of cells remained alive after treatment with different concentrations of AMP, which indicated that AMP was not toxic to RAW264.7 cells (Additional file 1: Fig. S4). Furthermore, in order to ensure that the NPs did not hemolyze in the blood in vivo, the hemolytic effect of AMP was evaluated. It could be found that there was no hemolysis at the concentration of 0–32 μg/mL, while hemolysis gradually appeared when the concentration was higher than 32 μg/mL (Additional file 1: Fig. S5). The safe concentration (16 μg/mL) was confirmed and adopted in the following study.

### Intracellular uptake and transfection efficiency of siRNA

**Fig. 4:**
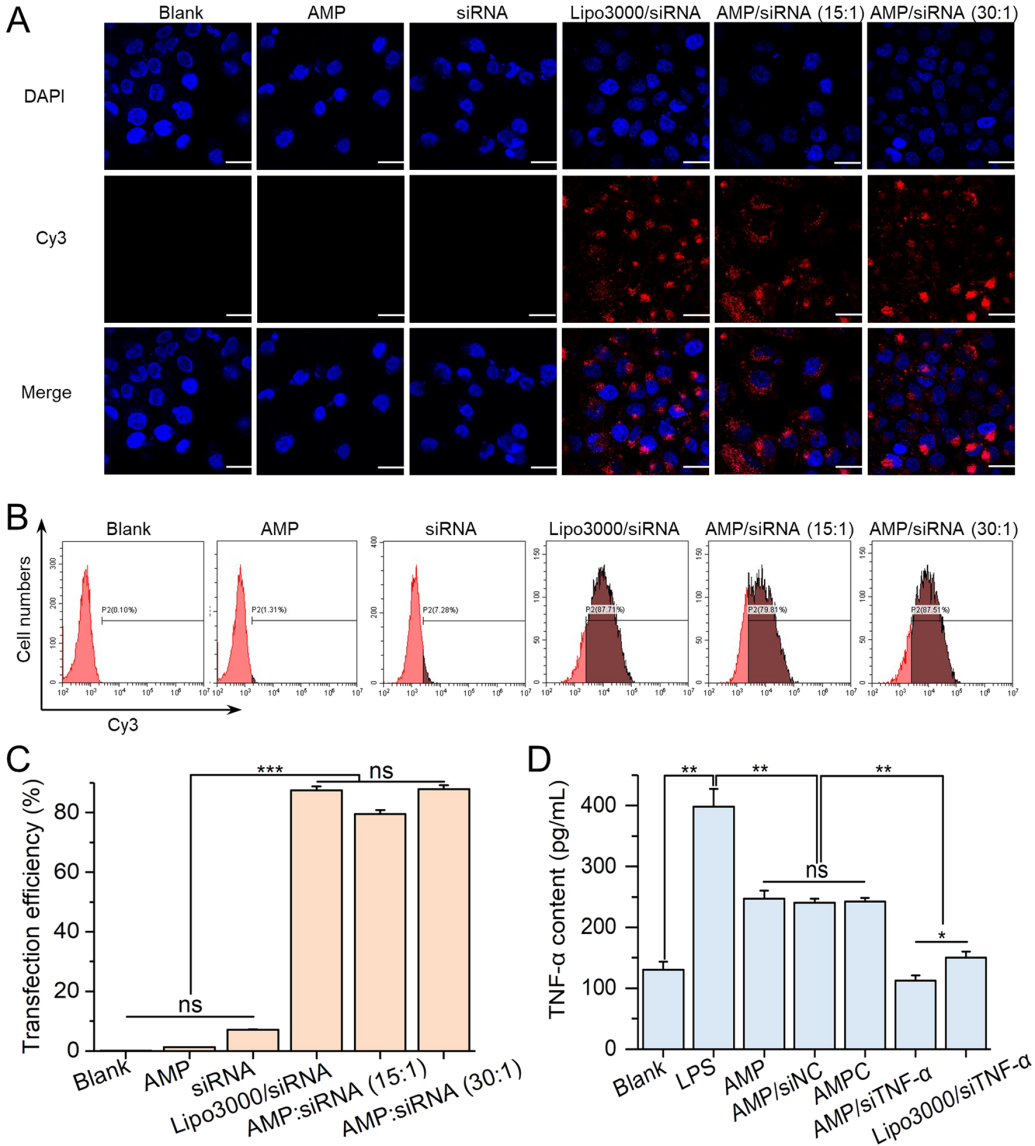
AMP-mediated siRNA transfection and gene silencing in LPS-induced RAW264.7 cells. **A** Fluorescence microscopy images of LPS-induced RAW264.7 cells treated with different formulations. Cell nuclei are stained with DAPI (blue). siRNA is labeled with Cy3. Thus, the intracellular siRNA will present red fluorescence signal. Scale bar = 20 μm. **B** Cell histograms and **C** Transfection efficiencies for evaluating the siRNA delivery effect by flow cytometry. **D** The expression levels of TNF-α protein in LPS-induced RAW264.7 cells treated with different formulations were evaluated by the TNF-α ELISA kit. The data are calculated by mean ± SD, n = 3 (*p < 0.05; **p < 0.01; ***p < 0.001). “ns” represents no significant difference

To demonstrate the siRNA delivery efficiency of the AMP, RAW264.7 cells were treated with AMP@siRNA, in which the siRNA was labeled with Cy3, and the intracellular fluorescence signal was monitored by an inverted fluorescence microscope. Non-treated, AMP, and naked Cy3-siRNA were used as the negative controls, while the commercialized siRNA transfection reagent, Lipo3000, served as the positive control. No Cy3 fluorescence signals (red) were observed from cells in the negative controls. In contrast, significant intracellular Cy3 fluorescence was observed in AMP@siRNA- and Lipo3000/siRNA-treated groups (Fig. 4A). Furthermore, quantitative analysis by flow cytometry revealed that the transfection efficiency (87.57%) of the group treated with AMP@siRNA at 30:1 (w:w) was almost the same as that (87.71%) of the positive control (Fig. 4B, C). And its mean fluorescence intensity was about twofold higher than that of the positive control (Additional file 1: Fig. S6). These results clearly suggested that AMP could be employed as an efficient carrier for intracellular siRNA delivery in macrophage cells.

### Gene silence efficiency of AMP@siTNF-α on LPS induced-macrophages

The efficiency of AMP-mediated siTNF-α delivery to knockdown TNF-α expression was evaluated by qRT-PCR. In LPS-induced RAW264.7 cells, the transcription level of TNF-α mRNA was up-regulated by 5.9-fold and the secreted TNF-α protein in medium supernatant increased by threefold compared with non-treated cells (Additional file 1: Fig. S7, Fig. 4D). After being treated with AMP, AMP@siNC and AMPC, the expression of TNF-α mRNA and protein decreased significantly, by 1.6-fold, which might be related to the anti-inflammatory properties of the released Ag [46, 47]. Moreover, their expression decreased most significantly (14.9-fold) in the group treated with AMP@siTNF-α than that of the positive (Lipo3000@siTNF-α) control (6.2-fold) (Additional file 1: Fig. S7). This indicated that siTNF-α was successfully and efficiently transfected to cells.

### Antibacterial activity of AMPC on *E. coli* and *S. aureus*

**Fig. 5:**
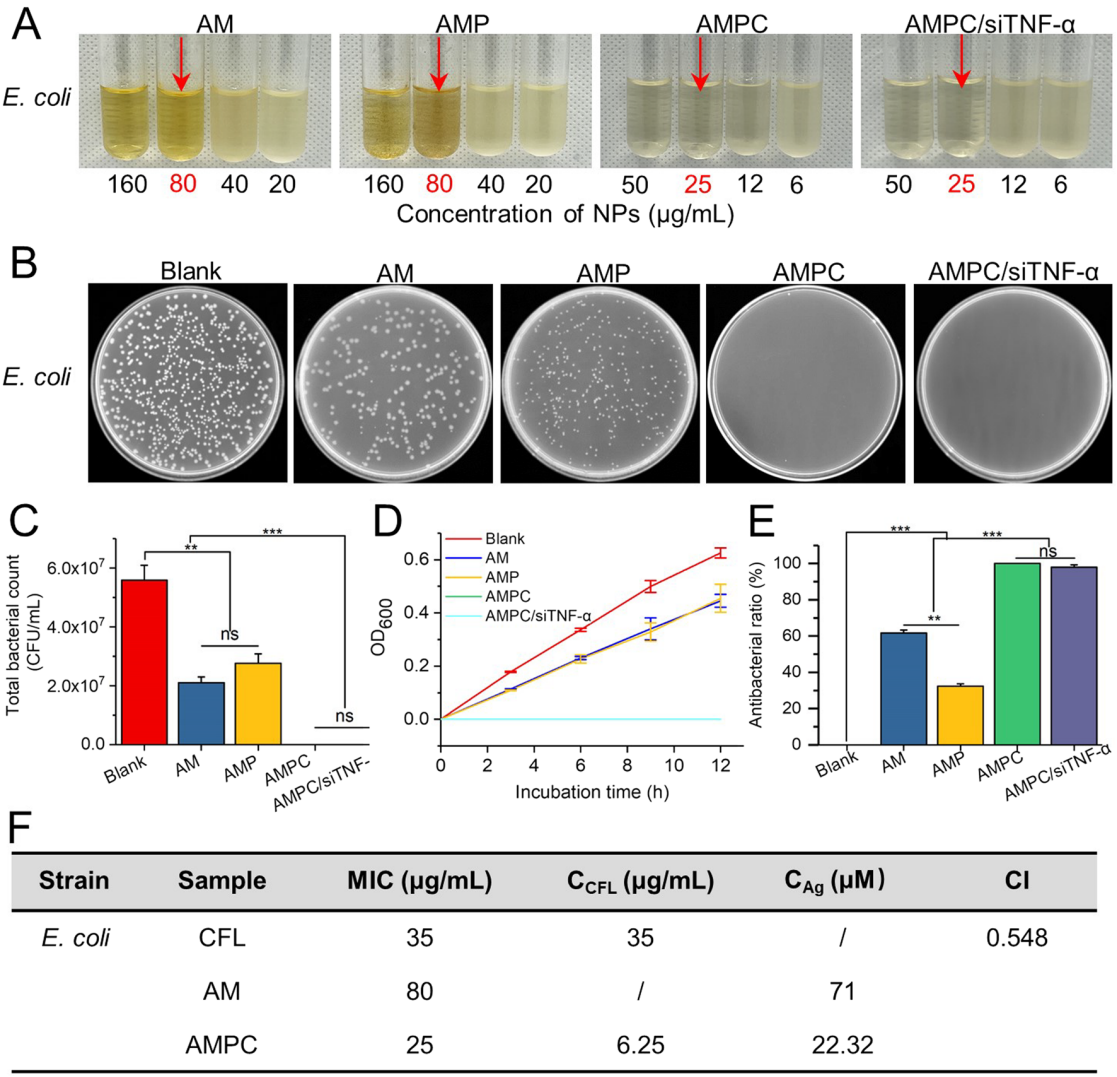
In vitro evaluation of the antibacterial activity of NPs. **A** The turbidity observation of *E. coli* in LB medium treated with different concentrations of formulations (AM, AMP, AMPC, and AMPC/siTNF-α). The MICs of the sample are marked with a red arrow. **B** The photographs of *E. coli* colony on agar plates, **C** quantitative bacterial colonies densities based on **B**, **D** growth curve, and **E** antibacterial ratio of *E. coli* in logarithmic growth period treated with different NPs for 12 h. The data represent mean ± SD, n = 3 (**p < 0.01; ***p < 0.001). “ns” represents no significant difference. **F** MIC of different antibacterial nanoplatforms for *E. coli* and the calculated CI value

To evaluate the in vitro antibacterial activity of different NPs, as Gram-negative and positive representative bacteria, *E. coli* and *S. aureus* were selected as a model, respectively [48]. The MICs of different NPs against the two bacteria were measured. The MICs of AM for *E. coli* and *S. aureus* were confirmed at 80 μg/mL and 120 μg/mL, respectively (Fig. 5A, Additional file 1: Fig. S8), which were comparable with the previous reports [16, 49]. The antibacterial ability of AM is related to the cell wall composition of the two bacteria. The wall of Gram-positive bacteria is mainly composed of peptidoglycan, which is more tenacious and can protect the plasma membrane from the attack of NPs. In addition, the wall of Gram-negative bacteria is mainly composed of peptidoglycan, lipoprotein, and phospholipid layer, which is relatively loose [50, 51]. The MICs of AMP against both strains were the same as those for AM, indicating that PEG-g-PEI did not affect the growth of bacteria (Fig. 5A, Additional file 1: Fig. S8). Moreover, after loading antibiotic CFL with AMP, the MICs (25 μg/mL) against *E. coli* and *S. aureus* decreased by 3.2-fold and 4.8-fold, respectively, which were the same as the MICs of AMPC/siTNF-α. This revealed that AMPC was more effective in the killing of *E. coli* and *S. aureus* and siTNF-α had no antibacterial activity. The most widely accepted explanation of the results was that the released Ag^+^ could interact with the thiol group in proteins on cell membranes to affect the vitality of bacterial cells by inhibiting DNA replication, which would play a synergistic antibacterial role with CFL [52].

According to the results of MICs, the different NPs with 50 μg/mL were chosen to explore their effect on anti-bacteria. After being cultured for 12 h at 37 °C, different NPs-treated bacterial liquid was coated on culture plates, and then quantitative bacterial colony densities were also evaluated. There were no colonies formed after AMPC treatment. Compared with the control group, the AM- and AMP-treated groups had fewer colonies (Fig. 5B, C, Additional file 1: Figs. S9, S10). The growth curves also showed that the OD values of the controls increased almost linearly with the increase of culture time and reached about 0.6 when cultured for 12 h (Fig. 5C, Additional file 1: Fig. S10). After treatment with different NPs, the treatment groups showed different degrees of inhibition of the two bacteria. Compared with the control, AM- and AMP-treated groups, in the whole culture process, the AMPC- and AMPC/siTNF-α treated group showed the same stronger antibacterial ability, and the OD value was the lowest (about 0) at 12 h (Fig. 5D, Additional file 1: Fig. S11). In addition, after different treatments, the antibacterial ratio was also measured. The antibacterial ratio of the AMPC- and AMPC/siTNF-α treated groups reached about 100% (Fig. 5E, Additional file 1: Fig. S12). Moreover, the synergistic effect in this system is also verified by the CI value of 0.548 (< 1) and 0.425 (< 1) for *E. coli* and *S. aureus*, respectively, demonstrating that AMPC had the excellent synergistic antibacterial effect and siTNF-α had no antibacterial activity (Fig. 5F, Additional file 1: Table S1).

### Efficiency of the AMPC@siTNF-α in promoting wound healing

**Fig. 6:**
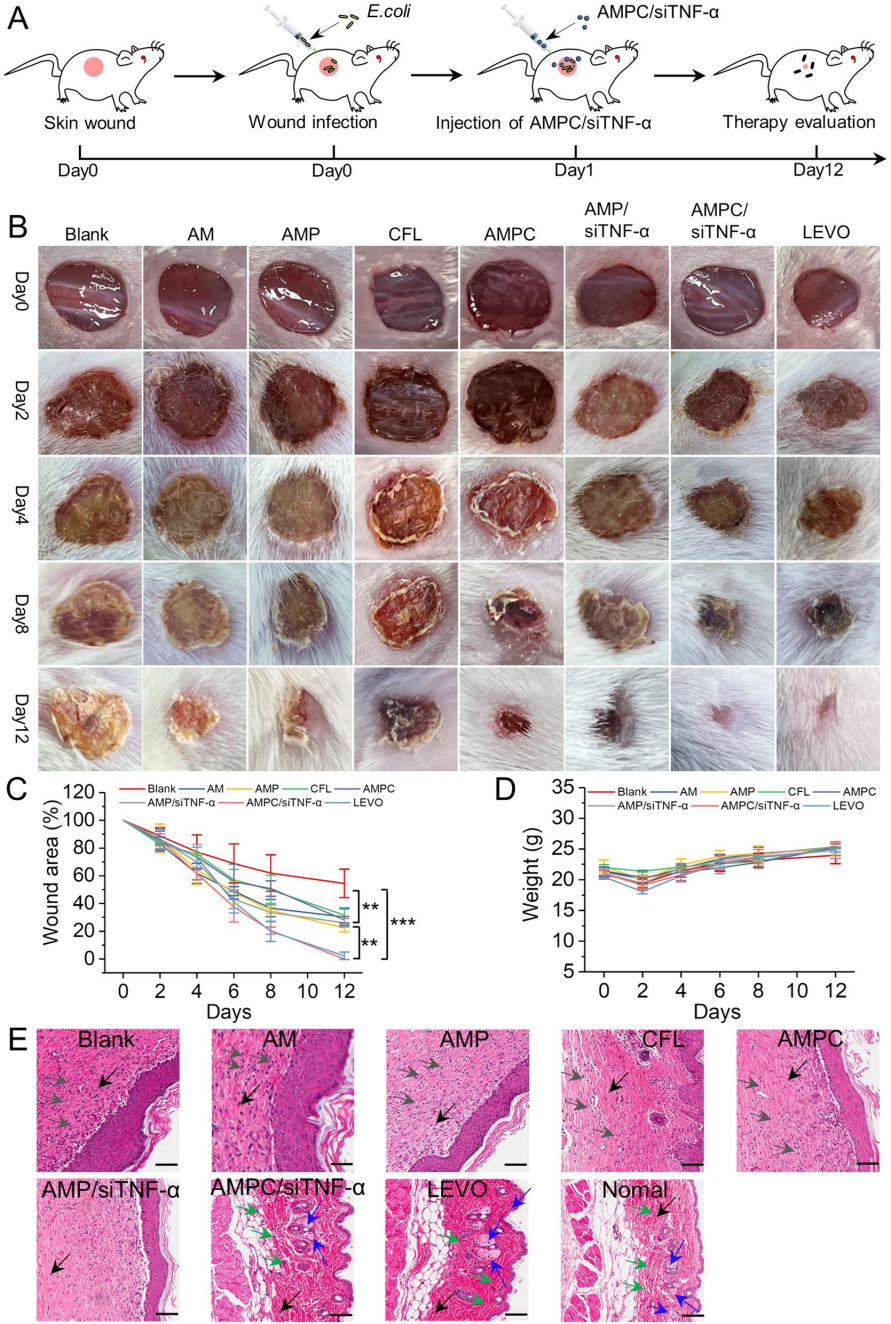
In vivo wound healing efficacy after being treated with different formulations. **A** The schematic diagram for the in vivo treatment evaluation procedure. **B** The photographs of *E. coli-* infected skin wound images treated with AM, AMP, CFL, AMPC, AMP/siTNF-α, AMPC/siTNF-α, and LEVO. **C** Closed area ratio of infected wounds (**p < 0.01; ***p < 0.001). **D **The change profiles of mouse weight treated with different formulations. **E** Histological graphs of skin tissue by H&E staining. Scale bar = 100 μm. Black, gray, green, and blue arrows indicate cell nucleus, neutrophils and inflammatory cells, fibroblasts, and sebaceous glands, respectively

Bacterial infection is the most severe interference factor in impeding wound healing. Excessive inflammation will destroy the residual epithelial tissue, resulting in collagen metabolism disorder and wound festering [53]. To evaluate the therapeutic effect of AMPC@siTNF-α in vivo, as illustrated in Fig. 6A, after the establishment of the wound model infected by *E. coli*, different NPs were placed on the wound surface. The whole course of treatment was completed within 12 days. As shown in Fig. 6B and C, the wounds in the control group showed obvious inflammation during the treatment. In contrast, the wound treated with AMPC@siTNF-α had no inflammation, scabs were formed after 8 days of treatment, and the wound healing completed close to 100% after 12 days, similar to the positive control (LEVO-treated). Remarkably, the AMPC@siTNF-α treatment exhibited the best wound healing effect. Furthermore, no weight loss was observed in all groups throughout the course, eliminating the security concern to the AMPC@siTNF-α (Fig. 6D).

Wound healing is a complex and important physiological process in the human body, which involves the reduction of neutrophils and inflammatory cells, proliferation of fibroblasts, and occurrence of sebaceous glands [54]. H&E staining was used to evaluate the infected tissues after 12 days of treatment. As shown in Fig. 5E, the groups treated with AMPC and AMPC@siTNF-α showed almost complete healing as indicated by the more fibroblasts and sebaceous glands, and less neutrophils and inflammatory cells, which were almost the same as the normal skin. For unhealed wounds (blank, AM, AMP, AMPC, and AMP@siTNF-α-treated group), the tissue contained large numbers of neutrophils and inflammatory cells. These results indicated that AMPC@siTNF-α could rapidly promote wound healing through the synergistic effect of released CFL and siTNF-α.

In addition, the main organs (heart, liver, spleen, lung, and kidney) of the treated mice were further analyzed by H&E staining (Additional file 1: Fig. S13). No obvious pathological changes and organic damage were found in the pathological section. In conclusion, these results strongly indicated that the treatment strategy based on AMPC@siTNF-α not only effectively promoted wound healing, but also had good biosafety in vivo.

## Conclusions

In conclusion, a multifunctional nanoplatform of AMPC@siTNF-α has been successfully fabricated and proved to be effective for the treatment of *E. coli*-infected wounds both in vitro and in vivo. The combination of the inner Ag core and the mesoporous silica shell displays the controlled release of Ag^+^, antibiotics, and siRNA simultaneously. AMPC exhibits superior antibacterial activity in vitro due to its synergistic effect between Ag^+^ and CFL. AMP@siTNF-α can be efficiently internalized by macrophages and significantly reduce the expression of the pro-inflammatory factor TNF-α *in vitr*o. In the in vivo wound infection model, the *E. coli* infected wound rapidly disappears after treatment with AMPC@siTNF-α, which is sixfold faster than that of the negative control and 2.5-fold faster than that of the single treatment group (AMPC- and AMP@siTNF-α-treated). Importantly, the nanoplatform has negligible toxicity with negligible side effects on mice during the test. This study strongly indicates a promising potential of AMPC@siTNF-α as a synergistic and safe therapeutic agent for clinical wound infections.

## Supplementary Information

**Additional file 1: Fig. S1.** The UV–vis spectra of ciprofloxacin (CFL) and Ag from the AMPC. **(A)** Absorption peaks of CFL with different concentrations at 275 nm. The standard curves of **(B)** CFL and **(C)** AMPC. **Fig. S2.** The encapsulation efficiency of CFL into AMP. **Fig. S3.** Agarose gel electrophoresis of AMPC@siNC. **Fig. S4.** Cell viability after the incubation of AMP at different concentrations with RAW264.7 cells. BLK represents the untreated cells. The data represent as means ± SD, n = 6. **Fig. S5.** The hemolysis result of AMP at different concentrations. The data represent as means ± SD, n = 6. **Fig. S6.** Average fluorescence intensities for evaluating the siRNA delivery effect by flow cytometry. The data are mean ± SD, n = 3 (*, *p* < 0.05; **, *p* < 0.01; ***, *p* < 0.001). “ns” represents no significant difference. **Fig. S7.** The expression of TNF-α mRNA in LPS-induced RAW 264.7 cells treated with different formulations by qRT-PCR. The data are mean ± SD, n = 3 (*, *p* < 0.05; **, *p* < 0.01; ***, *p* < 0.001). “ns” represents no significant difference. **Fig. S8** The turbidity observation of *S. aureus* in LB medium treated with different concentrations of formulations (AM, AMP, AMPC, and AMPC/siTNF-α). The MICs of the sample are marked with a red arrow. **Fig. S9** The photographs of the *S. aureus* colony on agar plates with different treatments for 12 h. **Fig. S10.** Quantitative bacterial colonies densities based on **Fig. S9** after different treatments for 12 h. The data represent mean ± SD, n = 3 (*, *p* < 0.05; **, *p* < 0.01; ***, *p* < 0.001). “ns” represents no significant difference. **Fig. S11.** Growth curve of *S. aureus* in logarithmic growth period treated with different NPs. **Fig. S12.** Antibacterial ratio of *S. aureus* after different treatments for 12 h. The data represent mean ± SD, n = 3 (*, *p* < 0.05; **, *p* < 0.01; ***, *p* < 0.001). “ns” represents no significant difference. **Fig. S13.** In vivo biosafety evaluation of NPs. H&E staining of histological sections including heart, liver, spleen, lung, and kidney of mice after 12 days of different treatments. LEVO represents levofloxacin. Scale bar = 100 μm. **Table S1.** MIC of different antibacterial nanoplatforms for *S. aureus* and the calculated CI value

## Data Availability

The data are all available upon request.
