# Supplementary material for: Mesoporous silica-coated silver nanoparticles as ciprofloxacin/siRNA carriers for accelerated infected wound healing
Source: J Nanobiotechnology. 2022 Aug 23;20:386. doi: 10.1186/s12951-022-01600-9 (PMC9400313; doi:10.1186/s12951-022-01600-9)
Supplement: Supplementary file 1 — Additional file 1: Fig. S1. The UV–vis spectra of ciprofloxacin (CFL) and Ag from the AMPC. (A) Absorption peaks of CFL with different concentrations at 275 nm. The standard curves of (B) CFL and (C) AMPC. Fig. S2. The encapsulation efficiency of CFL into AMP. Fig. S3. Agarose gel electrophoresis of AMPC@siNC. Fig. S4. Cell viability after the incubation of AMP at different concentrations with RAW264.7 cells. BLK represents the untreated cells. The data represent as means ± SD, n = 6. Fig. S5. The hemolysis result of AMP at different concentrations. The data represent as means ± SD, n = 6. Fig. S6. Average fluorescence intensities for evaluating the siRNA delivery effect by flow cytometry. The data are mean ± SD, n = 3 (*, p < 0.05; **, p < 0.01; ***, p < 0.001). “ns” represents no significant difference. Fig. S7. The expression of TNF-α mRNA in LPS-induced RAW 264.7 cells treated with different formulations by qRT-PCR. The data are mean ± SD, n = 3 (*, p < 0.05; **, p < 0.01; ***, p < 0.001). “ns” represents no significant difference. Fig. S8 The turbidity observation of S. aureus in LB medium treated with different concentrations of formulations (AM, AMP, AMPC, and AMPC/siTNF-α). The MICs of the sample are marked with a red arrow. Fig. S9 The photographs of the S. aureus colony on agar plates with different treatments for 12 h. Fig. S10. Quantitative bacterial colonies densities based on Fig. S9 after different treatments for 12 h. The data represent mean ± SD, n = 3 (*, p < 0.05; **, p < 0.01; ***, p < 0.001). “ns” represents no significant difference. Fig. S11. Growth curve of S. aureus in logarithmic growth period treated with different NPs. Fig. S12. Antibacterial ratio of S. aureus after different treatments for 12 h. The data represent mean ± SD, n = 3 (*, p < 0.05; **, p < 0.01; ***, p < 0.001). “ns” represents no significant difference. Fig. S13. In vivo biosafety evaluation of NPs. H&E staining of histological sections including heart, liver, spleen, lung [file 12951_2022_1600_MOESM1_ESM.docx]

**Additional file 1**

**Mesoporous silica-coated silver nanoparticles as ciprofloxacin/siRNA carriers for accelerated infected wound healing**

Qiqi Liu^1,^ **^†^**, Ying Zhang^1,2,^ **^†^**, Jingkai Huang^3^, Zhourui Xu^1^, Xiang Li^1^, Jingyu Yang^1^, Haoqiang Huang^1^, Shiqi Tang^1^, Yujuan Chai^1^, Jinbo Lin^2^, Chengbin Yang ^1,*^, Jia Liu^2,*^, Suxia Lin^4,*^

^1^Guangdong Key Laboratory for Biomedical Measurements and Ultrasound Imaging, School of Biomedical Engineering, Health Science Center, Shenzhen University, Shenzhen 518060, China;

^2^Central Laboratory, The Second Affiliated Hospital, School of Medicine, The Chinese University of Hong Kong, Shenzhen, Guangdong, 518172, P. R. China & Longgang District People’s Hospital of Shenzhen;

^3^Dermatology department, Southern University of Science and Technology Hospital (SUSTech Hospital, Shenzhen 518055, China;

^4^Center of Assisted Reproduction and Embryology, The University of Hong Kong-Shenzhen Hospital, Shenzhen 518048, China;

***Correspondence:** [cbyang@szu.edu.cn](mailto:cbyang@szu.edu.cn), Tel.: +86-755-26932656; liujia870702@126.com, Tel.: +86-755-28932833; [linsx@hku-szh.org](mailto:linsx@hku-szh.org), Tel.: +86-755-86913333

† These authors contributed equally to this work.


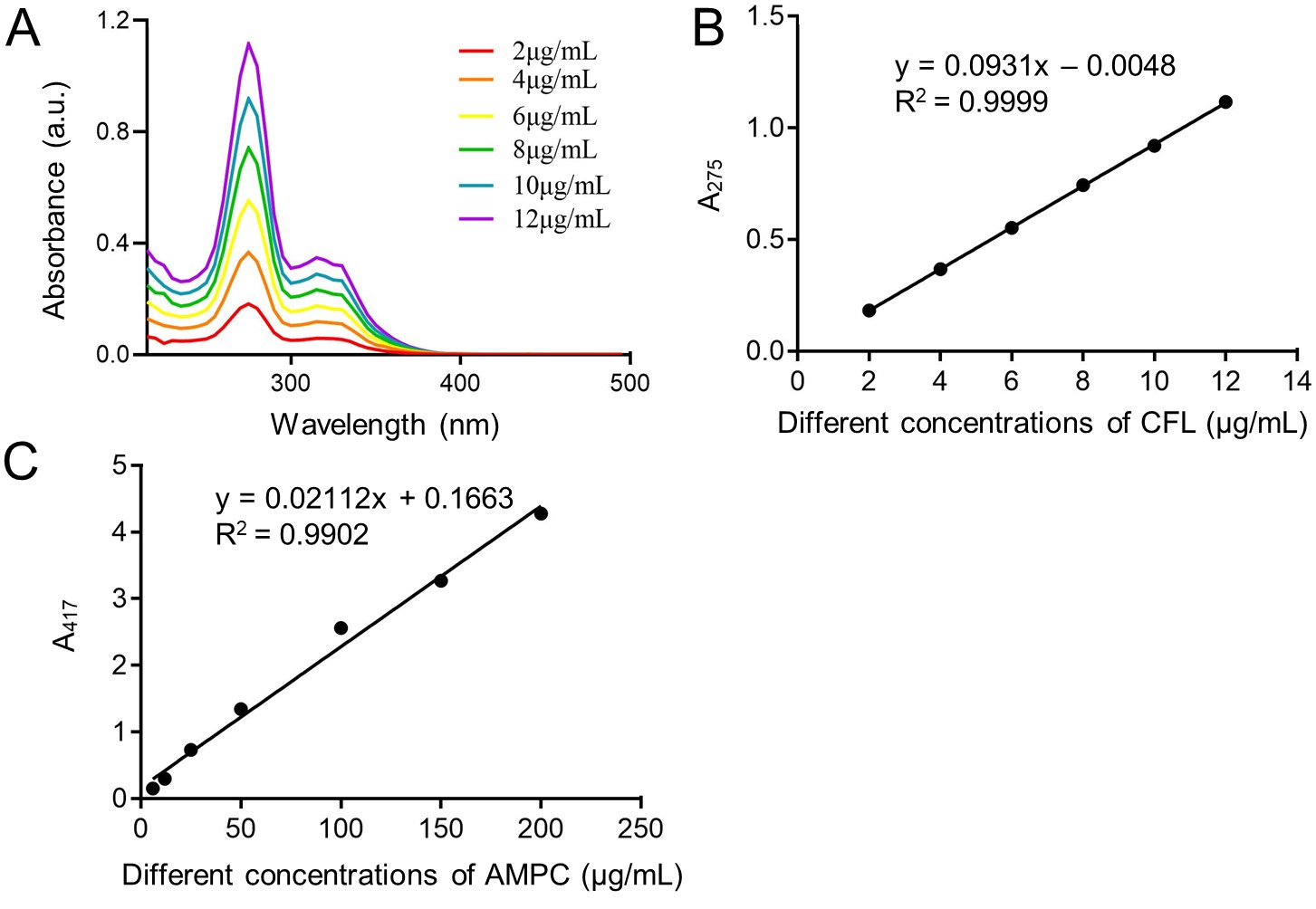


**Fig. S1** The UV-vis spectra of ciprofloxacin (CFL) and Ag from the AMPC. **(A)** Absorption peaks of CFL with different concentrations at 275 nm. The standard curves of **(B)** CFL and **(C)** AMPC.


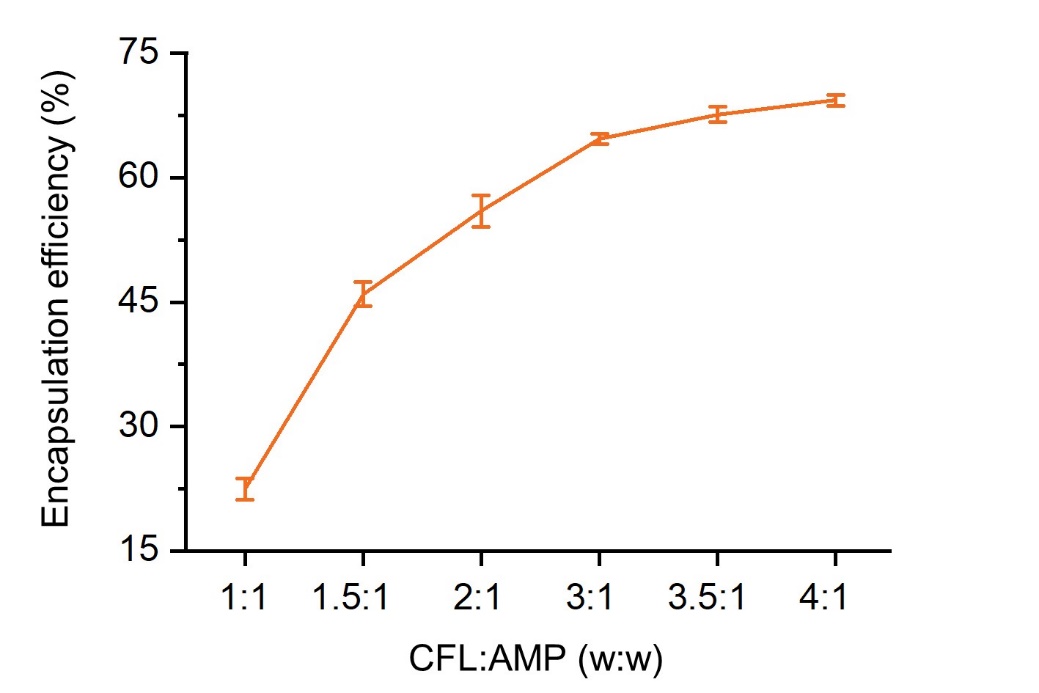


**Fig. S2** The encapsulation efficiency of CFL into AMP.


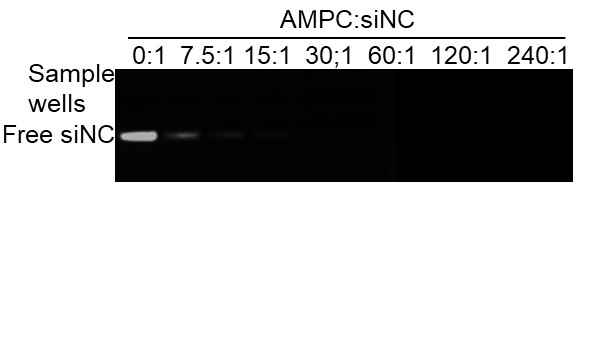


**Fig. S3** Agarose gel electrophoresis of AMPC@siNC.


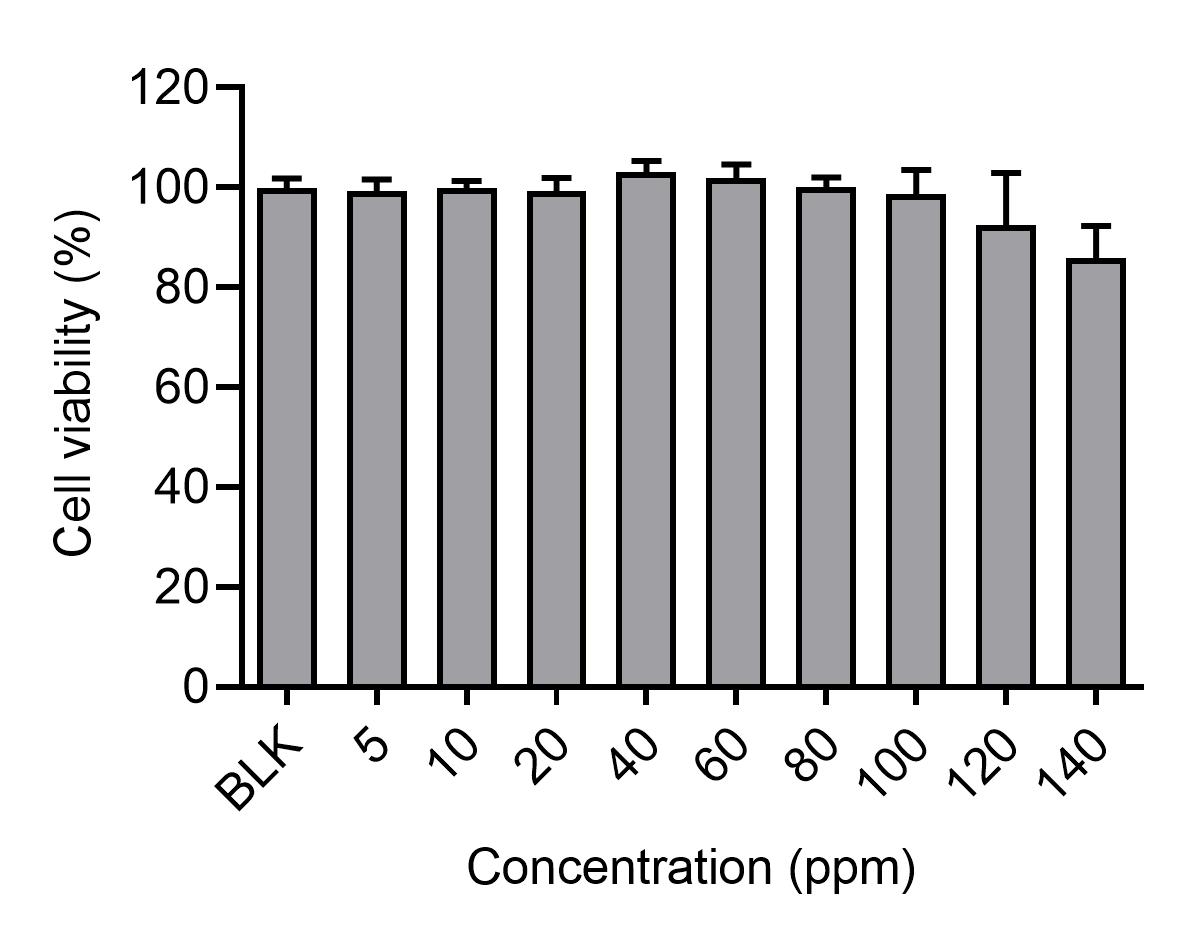


**Fig. S4** Cell viability after the incubation of AMP at different concentrations with RAW264.7 cells. BLK represents the untreated cells. The data represent as means ± SD, n = 6.

**
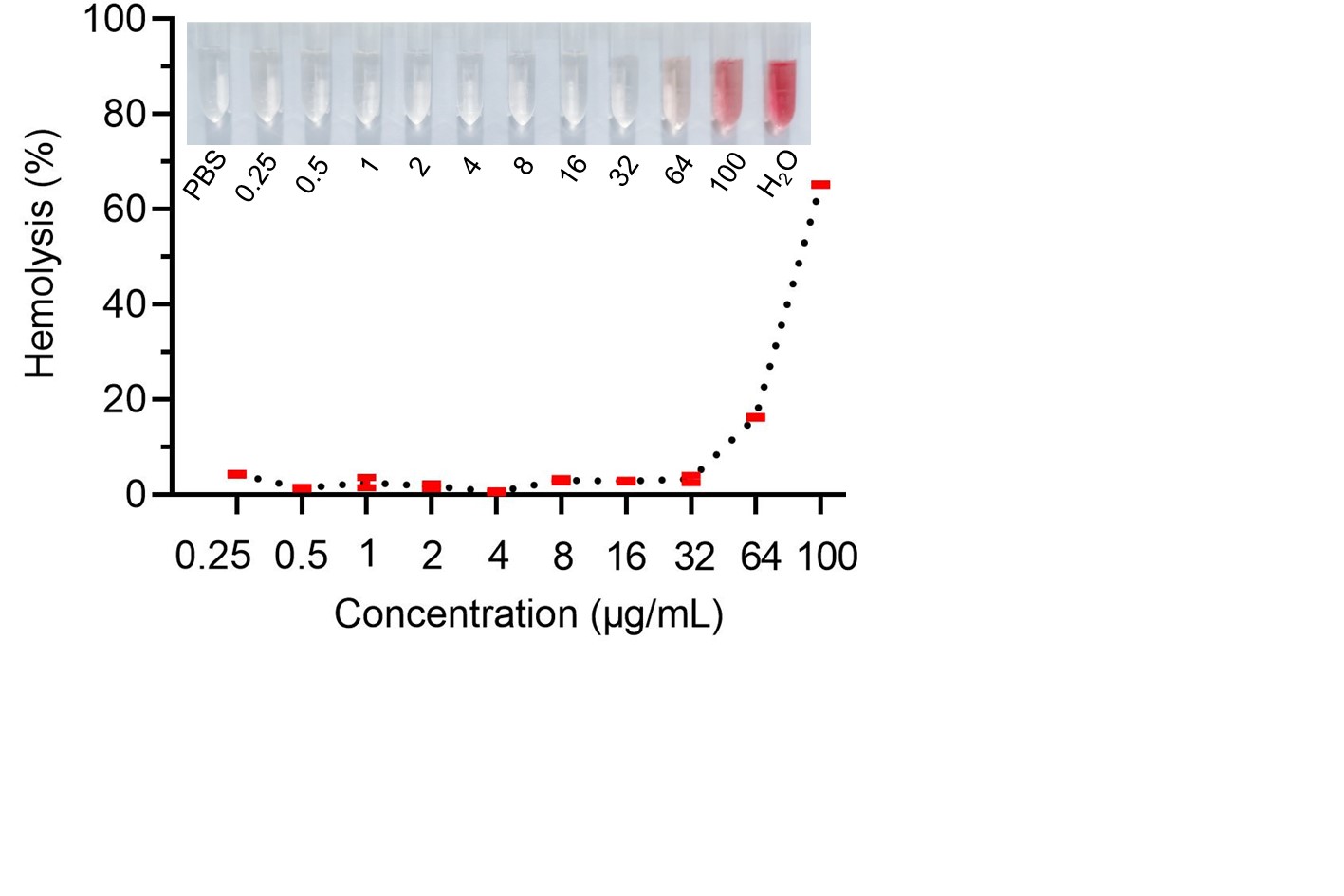
**

**Fig. S5** The hemolysis result of AMP at different concentrations. The data represent as means ± SD, n = 6.

**
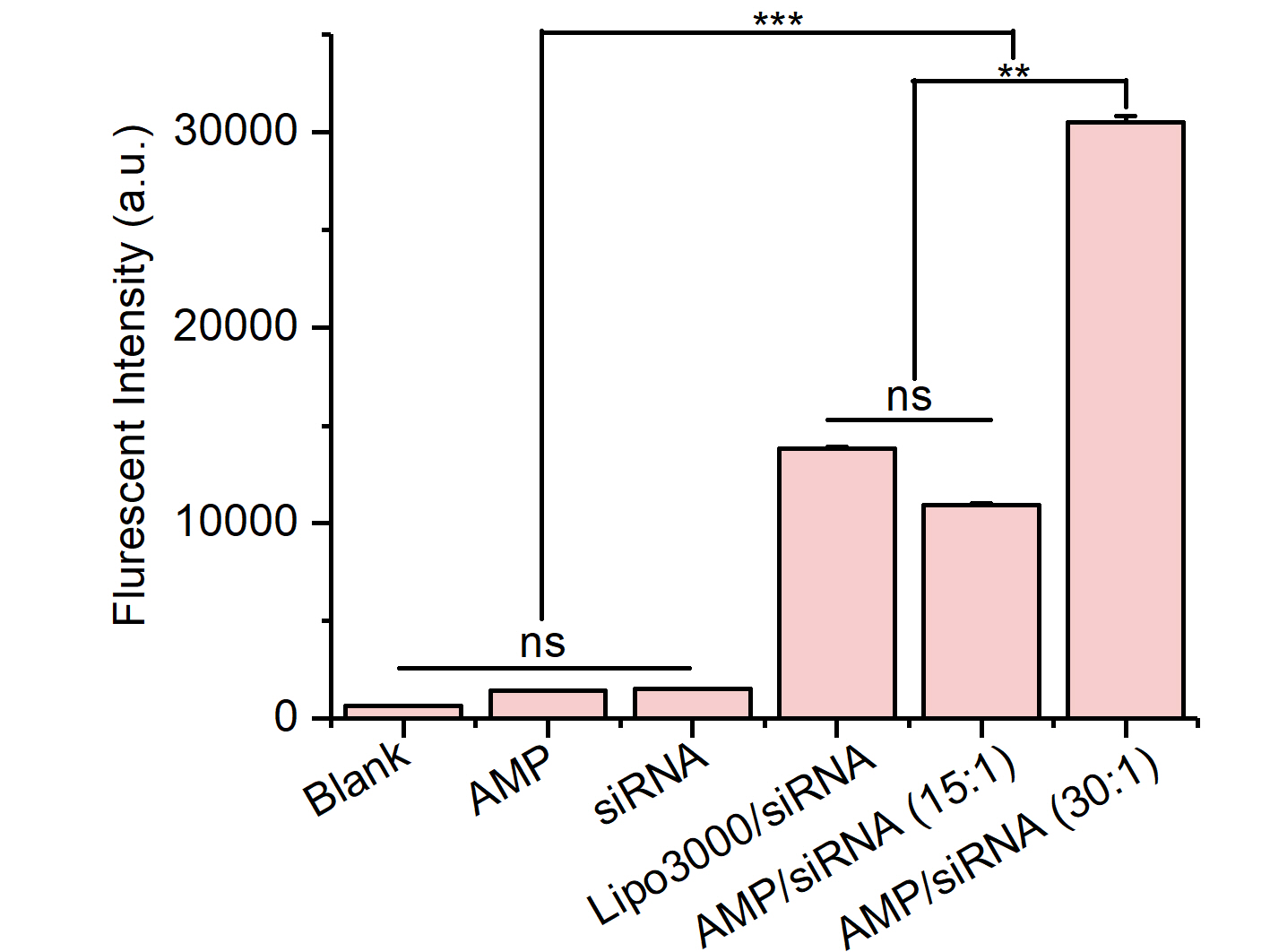
**

**Fig. S6** Average fluorescence intensities for evaluating the siRNA delivery effect by flow cytometry. The data are mean ± SD, n = 3 (*, *p* < 0.05; **, *p* < 0.01; ***, *p* < 0.001). “ns” represents no significant difference.

**
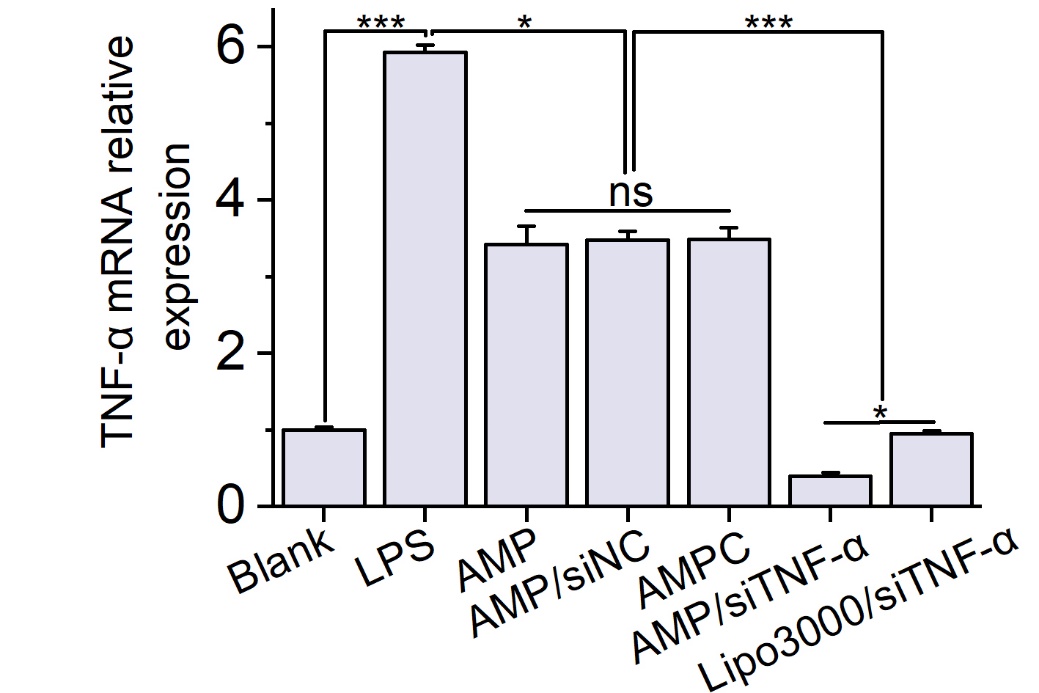
**

**Fig. S7** The expression of TNF-α mRNA in LPS-induced RAW 264.7 cells treated with different formulations by qRT-PCR. The data are mean ± SD, n = 3 (*, *p* < 0.05; **, *p* < 0.01; ***, *p* < 0.001). “ns” represents no significant difference.

**
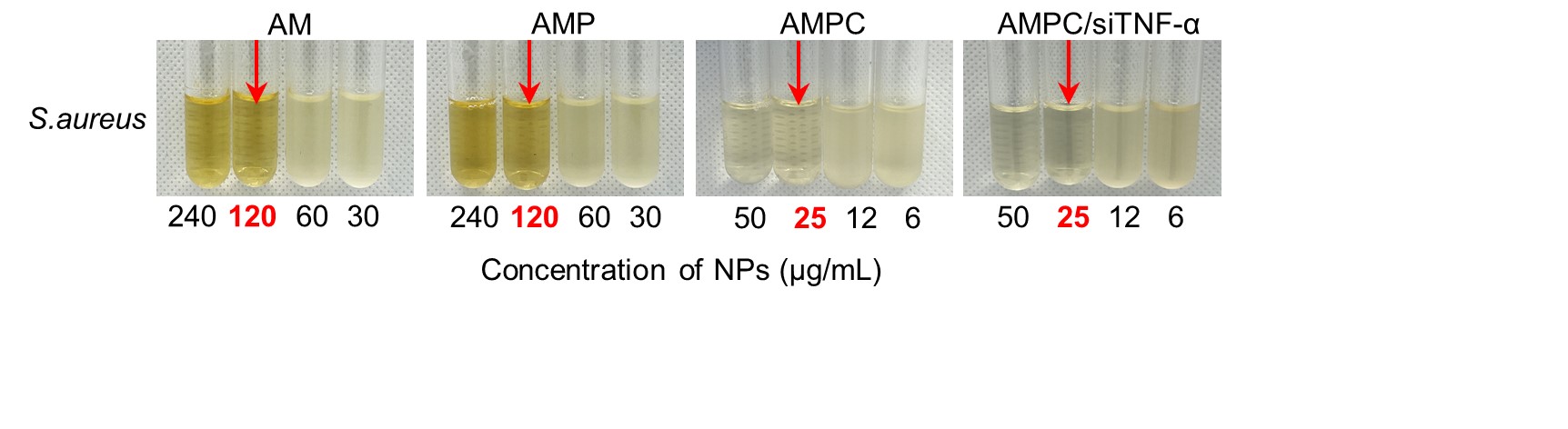
**

**Fig. S8** The turbidity observation of *S. aureus* in LB medium treated with different concentrations of formulations (AM, AMP, AMPC, and AMPC/siTNF-α). The MICs of the sample are marked with a red arrow.


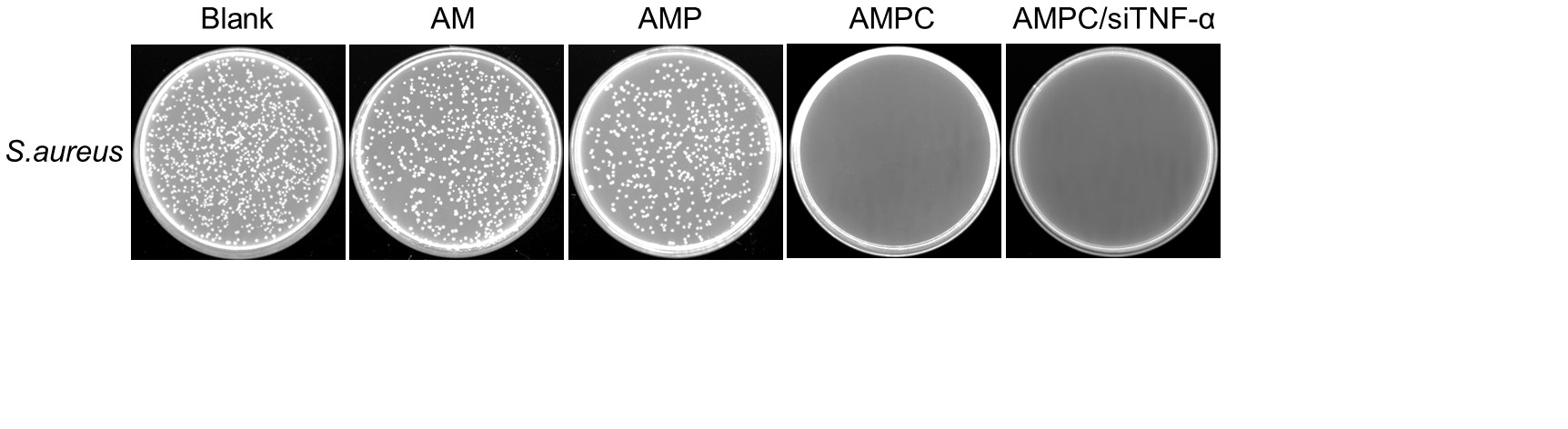


**Fig. S9** The photographs of the *S. aureus* colony on agar plates with different treatments for 12 h.

**
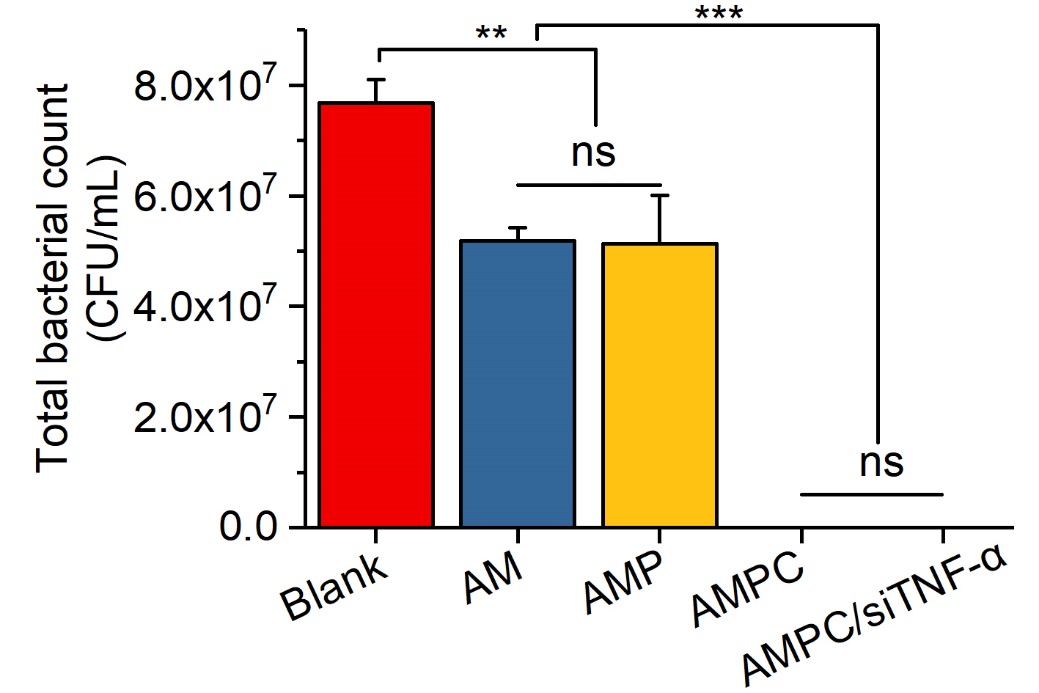
**

**Fig. S10** Quantitative bacterial colonies densities based on **Fig. S9** after different treatments for 12 h. The data represent mean ± SD, n = 3 (*, *p* < 0.05; **, *p* < 0.01; ***, *p* < 0.001). “ns” represents no significant difference.


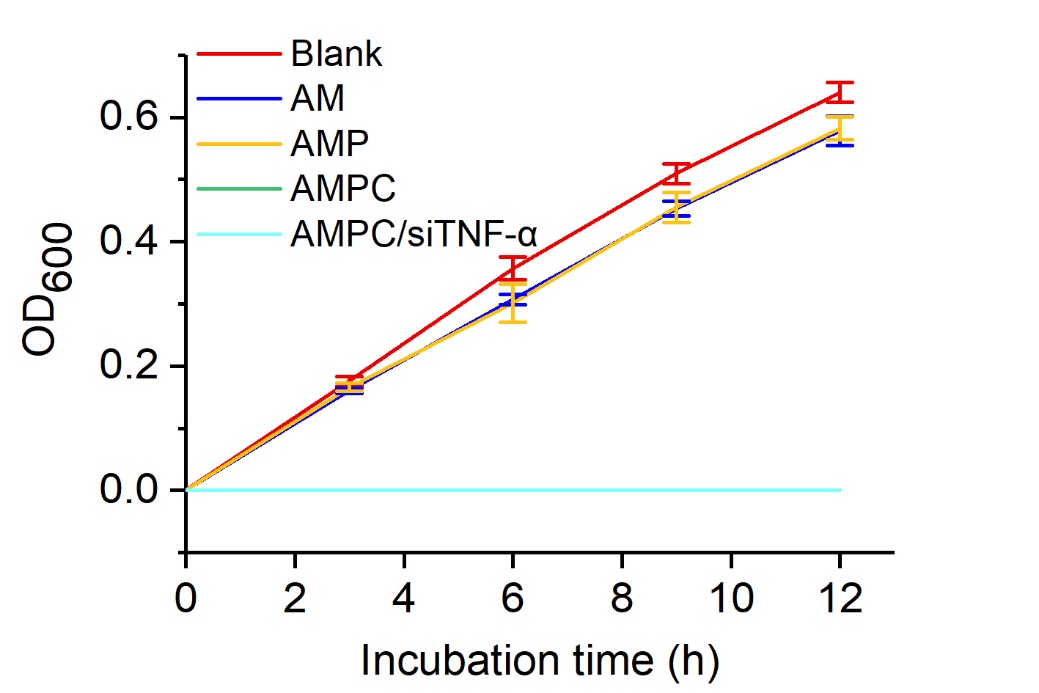


**Fig. S11** Growth curve of *S. aureus* in logarithmic growth period treated with different NPs.

**
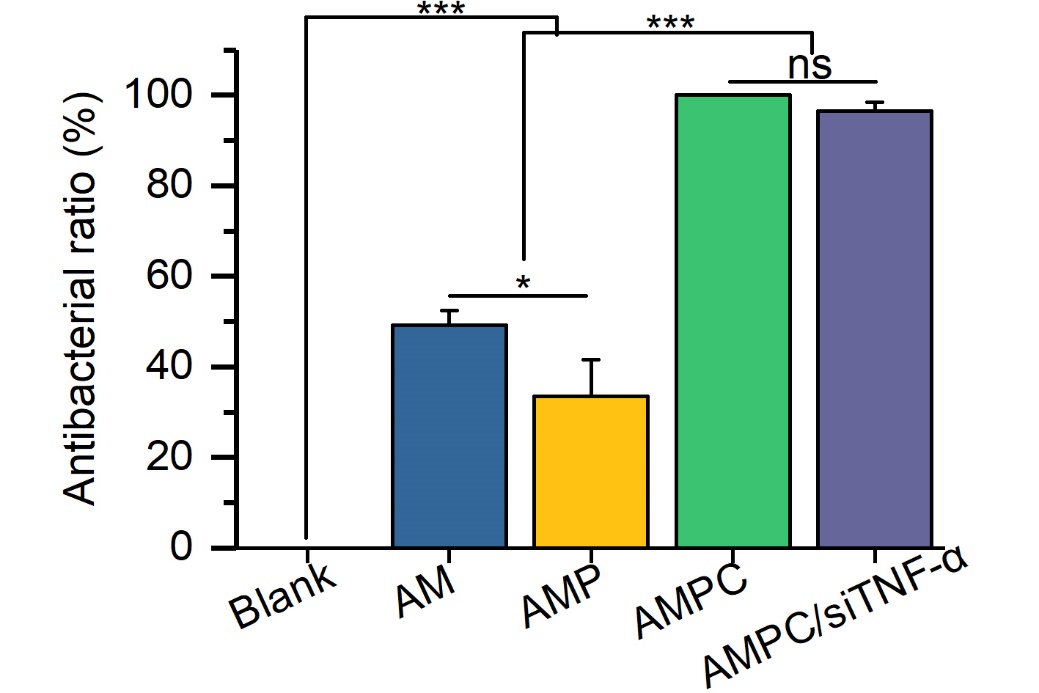
**

**Fig. S12** Antibacterial ratio of *S. aureus* after different treatments for 12 h. The data represent mean ± SD, n = 3 (*, *p* < 0.05; **, *p* < 0.01; ***, *p* < 0.001). “ns” represents no significant difference.


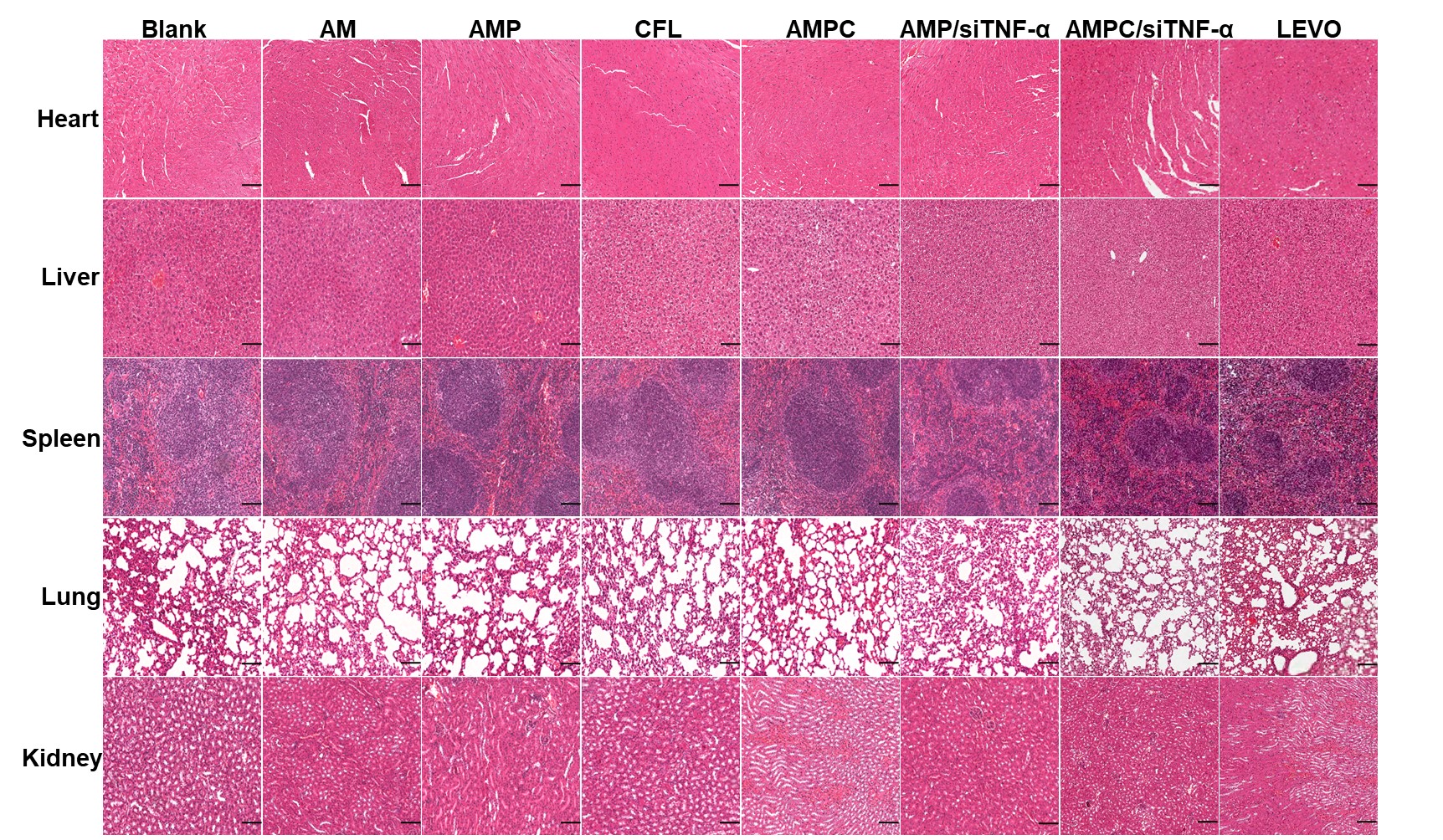


**Fig. S13** *In vivo* biosafety evaluation of NPs. H&E staining of histological sections including heart, liver, spleen, lung, and kidney of mice after 12 days of different treatments. LEVO represents levofloxacin. Scale bar = 100 μm.

**Table S1** MIC of different antibacterial nanoplatforms for *S. aureus* and the calculated CI value

| **Strain** | **Sample** | **MIC (μg/mL)** | **C_CFL_ (μg/mL)** | **C_Ag_ (μM)** | **CI** |
| --- | --- | --- | --- | --- | --- |
| *S. aureus* | CFL | 35 | 35 | / |  |
|  | AM | 120 | / | 106.2 | 0.425 |
|  | AMPC | 25 | 6.25 | 22.32 |  |
